# A Function-Centric Framework for Mitochondrial Quality Control

**DOI:** 10.3390/biom16081116

**Published:** 2026-07-30

**Authors:** Fulya Ozcan, Filip Vujovic, Ramin M. Farahani

**Affiliations:** School of Medical Sciences, University of Sydney, Sydney, NSW 2006, Australia; fulya.ozcan@sydney.edu.au (F.O.); filip.vujovic@sydney.edu.au (F.V.)

**Keywords:** mitochondria, quality control, signalling pathways, electron transport chain, cell cycle

## Abstract

Mitochondrial quality control (QC) comprises interconnected pathways that preserve organelle function by detecting damage and mediating repair, remodelling, or elimination of defective components. Although many sub-organellar QC mechanisms are well characterised, stress is often sensed first at the level of mitochondrial function rather than at individual molecular targets. Functional domains such as oxidative folding, bioenergetics, redox balance, pH, and thermogenesis act as sensory portals that detect perturbations and trigger adaptive reprogramming of mitochondrial activity. In this perspective, we provide a conceptual perspective for mitochondrial QC as a mechanistically integrated network, emphasising how changes in these functional states couple diverse QC modules—including proteases, antioxidant systems, mitochondrial dynamics, mitophagy, and mitochondrial-derived vesicles—into a unified surveillance system. We propose that primary stressors, such as redox imbalance, are progressively converted into secondary stress signals, including reactive oxygen species accumulation, membrane depolarisation, metabolite redistribution, and altered lipid or nucleic-acid structure. These secondary signals propagate across mitochondrial and cytosolic compartments, amplifying QC by coordinating the engagement of repair, remodelling, and organelle-elimination pathways. This cascading transformation of stress signals not only limits the impact of the initial insult but also enhances adaptive capacity by driving synergistic deployment of QC processes across multiple mechanistic layers.

## 1. Introduction

### 1.1. Mitochondrial Energetics: Underpinnings of an Organellar Stress Nexus

Mitochondria operate in a thermodynamic regime that favours the emergence of abiotic stressors and disruption of organellar homeostasis, including lipid peroxidation, protein misfolding, and mitochondrial DNA damage. While still debated [1], evidence from fluorescence-based thermometry suggests that, under conditions of high respiratory coupling, mitochondrial temperature may approach ~50 °C, potentially up to 10 °C above the surrounding cytosol—which aligns with the elevated temperature optima of several respiratory chain enzymes [2,3,4]. Developmental systems further underscore the magnitude of mitochondrial thermal output; for example, zebrafish embryos at the two-cell stage have been estimated to exchange heat at ~30 nW per cell [5], an order of magnitude above typical cellular metabolic rates [6,7]. Such enhanced thermogenesis reflects synchronised activation of a poised electron transport chain (ETC) [8], driven in part by the provision of limiting components such as heme [9]. When the ETC is fully activated and the proton motive force (Δp) is largely dissipated, temperature spikes of ≈4.8 K/s can be achieved [10], making sustained intra-mitochondrial temperatures near 50 °C plausible under some conditions. As these temperatures approach or exceed the upper stability threshold of many organellar proteins [11], mitochondria must deploy multiple adaptive strategies to maintain function under chronic intra-organellar thermal stress [12,13,14].

Reactive oxygen species (ROS) represent a second prominent class of abiotic stressors generated as by-products of electron leakage from the ETC [15,16]. Over-reduction of ETC components (for example, a reduced coenzyme Q pool) and an increased proton gradient can render forward electron transfer thermodynamically unfavourable, promoting electron leak upstream of complex IV and partial reduction of O_2_ to superoxide anion [17,18]. Superoxide anion and the derivative ROS exhibit a dual nature, acting both as signalling mediators [19,20,21] and as damaging agents that drive undesirable oxidation reactions [22,23]. This duality [24] places mitochondria as major ROS producers whose function depends on finely tuned redox at the centre of redox-dependent quality control. For instance, import of many intermembrane space proteins requires their delivery in a reduced, unfolded state followed by oxidative folding via the mitochondrial disulfide relay system [25,26,27], making this pathway acutely sensitive to redox perturbations. Consequently, mitochondria host elaborate QC mechanisms that monitor and buffer deviations in redox state [28,29].

A third emergent stressor arises from the “currency state” of protons within the organelle. Proton flux through the mitochondrial inner membrane is central to ATP synthesis, and transient imbalances in H^+^ flux can shift matrix pH outside its physiological range [30]. For example, proton transport via the ADP/ATP carrier [31] or ROS-driven activation of uncoupling proteins [32] is expected to acidify the matrix by increasing proton influx, even as these processes relieve Δp and thereby attenuate ROS production. In this context, matrix acidification can be considered a secondary stressor generated by an adaptive response to a primary stressor (ROS). Rather than representing a simple unintended side effect or “loophole” in mitochondrial design, such transitions from primary to secondary stressors suggest that cascades of stress signals are intrinsic features of mitochondrial network topology that facilitate flexible adaptation to fluctuating demands.

### 1.2. Functional Reprogramming of Mitochondria: A Prelude to QC Mechanisms

**Figure 1:**
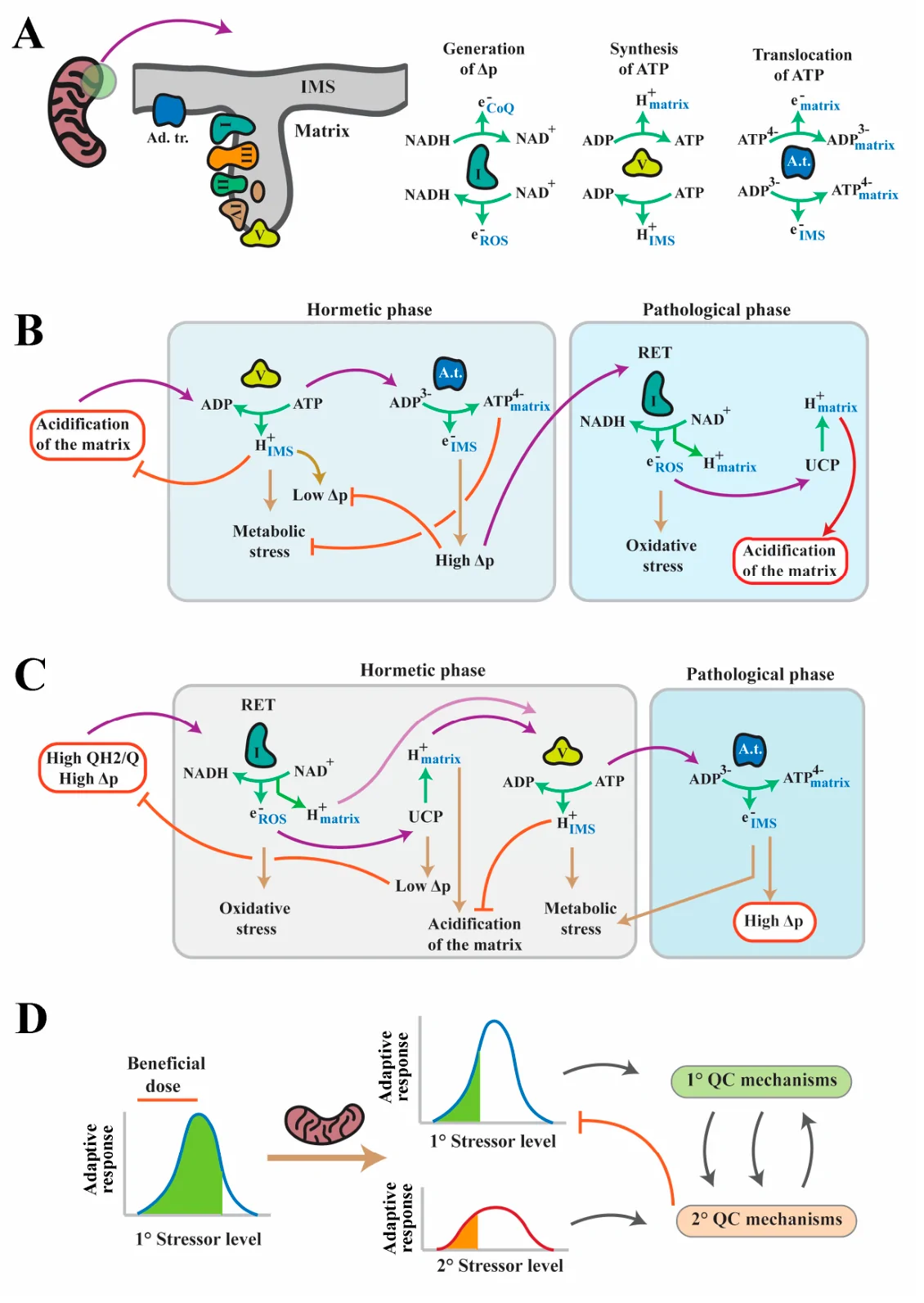
Mitochondrial conversion of primary stressors into secondary stress signals and integration into quality control cascades. (**A**) Schematic overview of the oxidative phosphorylation system illustrating bidirectional fluxes within the mitochondrial oxidative phosphorylation system. In functional mitochondria, each core component—the respiratory chain, protonmotive force (Δp), ATP synthase and adenine nucleotide translocator—operates as a bidirectional module whose net direction is set by prevailing homeostatic cues. (**B**) Adaptive rearrangement of oxidative phosphorylation in response to matrix acidification. A fall in matrix pH causes ATP synthase to operate in reverse, hydrolysing ATP and pumping protons out of the matrix to re-alkalinise the compartment, at the cost of increased ATP consumption. The accompanying rise in ADP^3−^ favours reverse transport by the adenine nucleotide translocator (ANT), increasing ATP^4−^ import and ADP^3−^ export to rebalance matrix nucleotides. This exchange helps correct the intra-mitochondrial ATP^4−^ deficit and hyperpolarise the inner membrane offsetting the collapsed Δp due to matrix acidification (hermetic phase). Beyond this hermetic phase, the sustained amplification of Δp could enhance ROS production, which in turn activates mitochondrial uncoupling proteins, leading to further acidification of the mitochondrial matrix (pathological phase). (**C**) Over-reduction of the coenzyme Q pool and increased Δp trigger reverse electron transport, leading to a cascade of events characterised by UCP-mediated proton influx, triggering acidification of the matrix and a reversal of ATP synthase activity to restore the physiological pH (hormetic response). However, accumulation of ADP^3−^ in long term would trigger a reversal of ANT flux with resultant increased ATP^4−^ import and ADP^3−^ export, further amplifying the underlying stressor of increased Δp (pathological response). (**D**) A schematic representation of the “cascade effect” model. Primary stressors exhibit a dose–response profile in which low levels elicit beneficial adaptation, whereas higher levels are damaging. Mitochondrial conversion of a primary stressor into one or more secondary stressors broadens the adaptive range by reducing the effective amplitude of the primary insult and generating additional, qualitatively distinct signals. These secondary stressors engage secondary QC mechanisms that operate alongside primary QC modules, creating a cascade-like network of partially non-overlapping, yet reciprocally reinforcing, mitochondrial quality control responses.

An intuitive way to represent mitochondrial function is as a bimodal switch built from interconnected bi-directional loops whose collective activity either supports ATP synthesis (ATP^on^) or favours ATP hydrolysis (ATP^off^) [8,33] (Figure 1A). Each loop can operate in two directions with distinct functional outputs. For example, the F_1_F_0_-ATP synthase catalyses proton influx from the intermembrane space (IMS) to the matrix to generate ATP, but under certain conditions it reverses to pump protons out of the matrix at the expense of ATP hydrolysis [33]. From this perspective, the states adopted by individual loops as the network toggles between ATP^on^ and ATP^off^ primarily serve to maintain or restore organellar homeostasis and can be at least partially uncoupled from the global ATP economy of the cell.

Under physiological conditions, F_1_F_0_-ATP synthase synthesises ATP by using the proton motive force generated across the inner membrane. When the mitochondrial matrix acidifies, the enzyme can switch to reverse mode and drive proton efflux to re-alkalinise the matrix, at the cost of increased ATP hydrolysis as demonstrated in anoxia-tolerant painted turtle cortical neurons [30] and in mammalian cells [31] (Figure 1B). This corrective response could potentially convert matrix acidification into a secondary metabolic stress by depleting ATP and elevating ADP. The resulting rise in the ADP:ATP ratio, and the amplified increase in AMP:ATP via adenylate kinase (AMP:ATP ∝ (ADP:ATP)^2^) [34], activates AMP-activated protein kinase (AMPK), a central sensor of cellular energy stress [35,36]. It can be argued that AMPK signalling would then alleviate ATP depletion by stimulating glycolysis, providing additional ATP that can be rationed back to mitochondria [37].

Within mitochondria, elevated ADP^3−^ promotes the reverse activity of adenine nucleotide translocator (ANT), increasing ATP^4−^ import and ADP^3−^ export [38,39] to rebalance matrix nucleotides. This not only offsets the ATP^4−^ deficiency within the organelle but also brings about mitochondrial hyperpolarisation due to the electrogenic nature of the exchange (net accumulation of −1 charge in the matrix per exchange event) [38]:

$${matrix\;ADP}^{3-}\overset{ANT}{\to }cytosol\;\;\;\;\;\;\;cytosol\;{ATP}^{4-}\overset{ANT}{\to }matrix\;\;\overset{Net\;gain}{\Rightarrow }\frac{1\left[e^{-}\right]}{cycle}$$

This hyperpolarised state would initially offset the reduction of Δp as a result of matrix acidification. Beyond this adaptive phase, an extended hyperpolarisation renders mitochondria prone to ROS generation via reverse electron transport (RET) at complex I [40] (Figure 1B). ROS could in turn activate mitochondrial uncoupling proteins [32], exacerbating the primary organellar stress of matrix acidification (Figure 1B). Together, these transitions illustrate how correcting a primary perturbation (matrix acidification) drives a cascade of secondary and tertiary stressors that successively redistribute the energetic burden across the network. Following this logic, a primary stressor can be defined as a deviation of mitochondrial functional parameters from its physiological range, whereas secondary and tertiary stressors emerge after the initial functional perturbation as responses to that event.

The same network architecture can potentially accommodate different primary stressors by engaging the loops in a different order. In an ETC that is highly reduced (elevated coenzyme QH_2_/Q ratio) and strongly polarised, forward electron transfer towards complex IV becomes thermodynamically disfavoured [41], promoting RET and bursts of ROS production at complex I [42] (Figure 1C). ROS could then activate uncoupling proteins [32], dissipating Δp and lowering ROS output, but at the cost of matrix acidification due to increased proton influx. This secondary acidification could potentially trigger reverse-mode ATP synthase to restore the matrix pH, again consuming ATP and propagating the cascade described above (Figure 1C). Thus, whether the primary insult is matrix acidification or excessive ROS/Δp, the mitochondrial network is expected to respond by re-ordering the same set of bidirectional loops to generate a characteristic “cascading transformation” of stressors that both constrain damage and redistribute stress.

$${pH}_{matrix}↓\overset{1^{{}^{\circ}}st.}{\to }\;Rev.\;ATP\;syn.\overset{2^{{}^{\circ}}st.}{\to }Rev.\;ANT\overset{3^{{}^{\circ}}st.}{\to }ROS↑$$

$$ROS↑\overset{1^{{}^{\circ}}st.}{\to }\;UCP↑/∆p↓\overset{2^{{}^{\circ}}st.}{\to }Rev.\;ATP\;syn.\overset{3^{{}^{\circ}}st.}{\to }Rev.\;ANT$$

While the proposed cascade effect arises naturally from the functional network topology of mitochondria, it is expected to yield an efficient pro-survival strategy that attenuates the impact of stressors and enables adaptation across multiple functional domains without compromising organellar viability. The impact of ROS on mitochondrial Lon protease illustrates why such multi-domain adaptation is advantageous. Lon recognises and degrades oxidised proteins in the matrix [43], but excessive ROS can impair its proteolytic activity, limiting its capacity to clear damaged substrates [44,45]. By partially converting oxidative stress into other stress modalities, the cascade dynamic helps keep ROS levels within a range that preserves Lon function and prolongs its contribution to mitochondrial protein quality control.

Mitochondrial conversion of stressors, framed as the “cascading transformation” in this review, is aligned to the broader well-established concept of hormesis [46] and mitohormesis [47]. Hormesis describes a biphasic response in which low doses of a stressor induce beneficial adaptations, whereas high doses become inhibitory or toxic. By redistributing a primary disturbance into secondary and tertiary stressors, mitochondria avoid saturating any single adaptive module and keep the interaction with the initial stressor within the beneficial arm of the hormetic curve (Figure 1D). In addition, emergent secondary and tertiary stressors can themselves elicit hormetic responses in other functional domains, thereby both complementing the adaptation to the primary stressor and recruiting additional quality control mechanisms.

Complementing this cascading transformation, mitochondrial quality control mechanisms form a surveillance network that restricts, repairs, or removes molecular damage induced by primary, secondary, and tertiary stressors. Returning to the example of RET-mediated oxidative stress, the metabolic stress generated by ATP hydrolysis is a bystander consequence of the response that restores matrix pH (Figure 1D). This secondary metabolic stress activates AMP-activated protein kinase (AMPK) via an increased AMP:ATP ratio, which in turn enhances mitochondrial antioxidant capacity by phosphorylating Forkhead box protein O1 (FoxO1) to drive Manganese Superoxide Dismutase (MnSOD) expression [48] and, via PGC-1α (Peroxisome proliferator-activated receptor gamma coactivator 1-α), by increasing MnSOD and NAD^+^-dependent deacetylase Sirt3 activity [49]. Thus, a second-tier stressor (metabolic stress) arising from the response to a primary insult (oxidative stress) engages a mitochondrial QC module that ultimately limits the scope of the original ROS challenge.

In this review, we examine mitochondrial QC mechanisms with an emphasis on how connectivity creates a surveillance network that operates as the counterpart to the cascading transformation of mitochondrial stressors. We argue that stressor-induced functional reprogramming of mitochondria not only provides an emergent reserve capacity to assemble this QC network without compromising organellar activity, but also actively reduces the effective burden imposed by mitochondrial stressors.

## 2. Mitochondrial Quality Control Mechanisms

### 2.1. Mitochondrial Proteostasis

Mitochondrial proteins are continually exposed to abiotic stressors that challenge their stability, including shifts in redox environment, increased thermal output, and changes in ionic conditions such as pH. Oxidative perturbations typically lead to selective modification of redox-sensitive residues [50], while elevated temperature and altered pH can destabilise protein conformation and folding [11]. In mitochondria, tight regulation of these stressors is particularly critical because the effects extend beyond mature folded proteins to encompass protein import and oxidative folding pathways.

The intermembrane space (IMS) contains some 150 resident proteins, and one third of IMS proteins of mitochondria in humans are predicted to be, or have been shown to be, substrates of the Mia40–Erv1 disulfide relay, the major oxidative folding system that stabilises conserved cysteine motifs via disulfide bond formation [51,52,53]. This relay is redox-vulnerable at multiple levels. First, efficient oxidative folding requires that precursor proteins arrive in the IMS in a reduced, unfolded state [54]; oxidation of critical cysteines in the cytosol is therefore expected to impede protein import and proper folding. Second, regeneration of oxidised relay components is coupled to the electron transport chain [55], as Erv1 passes electrons to cytochrome c and cytochrome c oxidase to re-oxidise Mia40 [56]. Consequently, changes in IMS redox state or impaired electron transport would be predicted to slow relay turnover and compromise the stability of newly imported proteins (for example, by limiting the availability of oxidised Mia40).

Proton concentration adds an additional layer of control. Mitochondrial protein import is strongly pH-dependent [57], with increasing proton concentration inhibiting translocation through TOM/TIM channels, such that import is significantly reduced at matrix-proximal pH values below ~6.5. Beyond import, proton abundance shifts the redox potential of proton-coupled reactions [58], while temperature likewise influences redox equilibria as demonstrated by findings in *Pyrococcus furiosus* [59] according to Nernst-type relationships, altering how a given level of ROS perturbs protein thiols and metal centres. These coupled dependencies on redox state, pH, and temperature help explain why mitochondria have evolved multiple, partially overlapping quality control and surveillance systems that buffer proteins against stress, stabilise folding and import pathways, and repair or eliminate damaged species. In the following sections, we consider these proteostasis mechanisms in two broad categories: protein-stabilising pathways and protein repair/elimination mechanisms.

### 2.2. Mitochondrial Heat Shock Proteins: Duality of Function and Divergence of Outcomes

**Figure 2:**
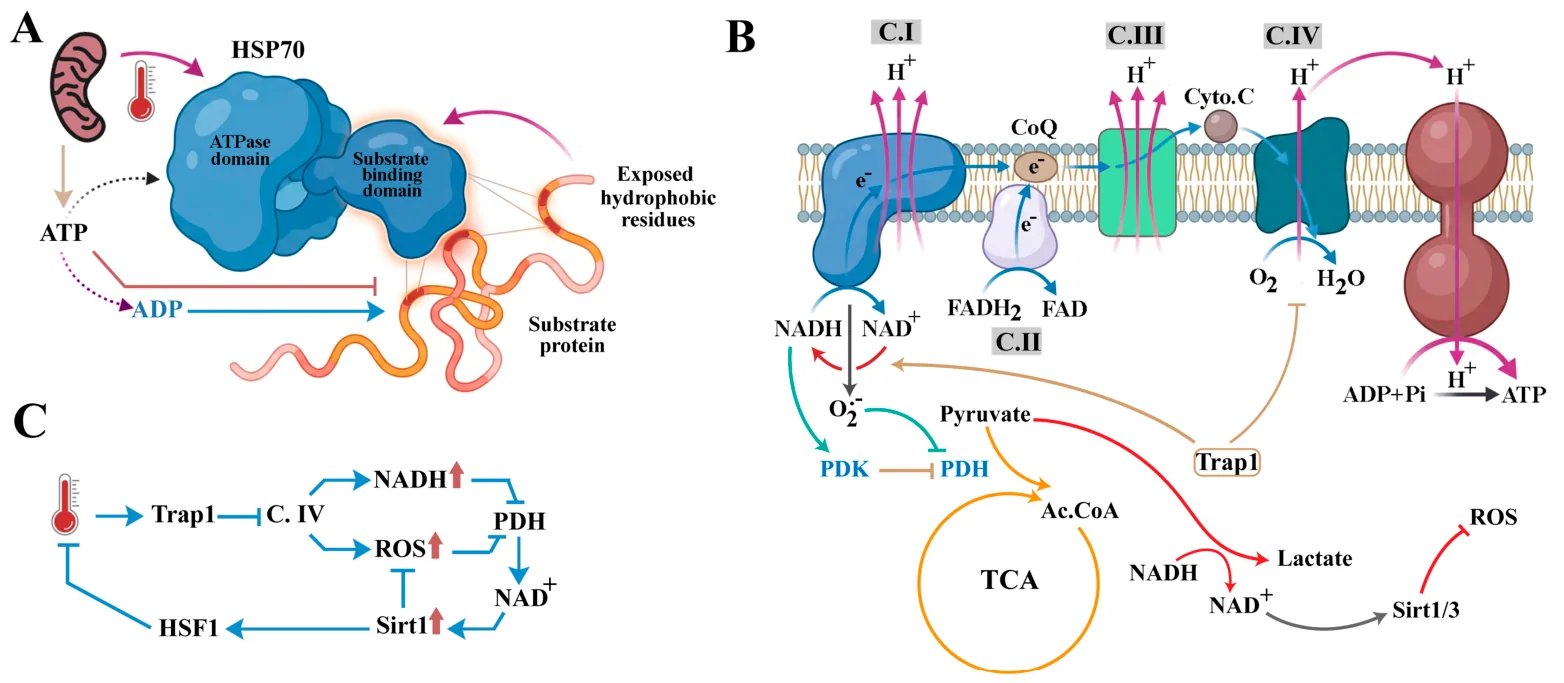
Heat-induced remodelling of mitochondrial chaperone networks and respiratory metabolism. (**A**) Schematic representation of HSP70-family chaperone activity. Heat-shock-dependent recruitment of HSP70 to client proteins under metabolic stress. During heat shock, enrichment of ADP in metabolically stressed mitochondria shifts HSP70 toward its high-affinity, ADP-bound state, stabilising interactions between the chaperone’s substrate-binding domain and exposed hydrophobic regions of unfolded proteins. In this way, elevated ADP acts as a co-signal that enables robust engagement of HSP70 with heat-denatured substrates, promoting their refolding or routing into downstream quality control pathways. (**B**) Integration of chaperone function with respiratory chain activity and central carbon metabolism. Electron transfer through complexes I–IV generates a proton gradient across the inner mitochondrial membrane and drives ATP synthesis, while electron leak gives rise to superoxide and other ROS. The mitochondrial chaperone Trap1 modulates this system by inhibiting complex IV, thereby promoting reverse electron flow and enhancing ROS production. The resulting ROS inhibit pyruvate dehydrogenase, diverting pyruvate toward anaerobic glycolysis, which regenerates NAD^+^ and activates Sirt1. In this way, Trap1-dependent tuning of respiratory flux and ROS signalling couples heat-induced chaperone activity to a Sirt1-mediated metabolic adaptation that supports survival under thermal stress. (**C**) Heat-driven Trap1–ROS–Sirt1 circuit supporting adaptation to thermal stress. Thermal shock activates the mitochondrial chaperone Trap1, which inhibits complex IV and thereby elevates NADH levels, promoting pyruvate dehydrogenase (PDH) inhibition via pyruvate dehydrogenase kinase (PDK). In parallel, reverse electron transport-derived ROS directly inactivate PDH. Together, these effects divert pyruvate toward anaerobic glycolysis, increasing the rate of NAD^+^ regeneration and enhancing Sirt1 activity. Activated Sirt1 then stimulates HSF1, reinforcing heat-shock responses and promoting cellular acclimation to thermal stress. Thus, ROS generated as a secondary stress signal contribute to the adaptive response to the primary stressor, heat.

Heat shock proteins (HSPs) are specialised molecular chaperones that recognise a broad range of substrate proteins and promote folding or refolding under destabilising conditions [60,61]. Many HSPs engage substrates through flexible, low-complexity regions [62] that also contribute to the assembly into oligomeric complexes [60]. Stress-induced disassembly of these oligomers exposes substrate-binding interfaces and permits engagement of misfolded proteins, thereby stabilising vulnerable substrates (Figure 2A). The most abundant mitochondrial chaperones are members of the HSP10, HSP60, and HSP70 families [63], with additional contributions from HSP90-family TRAP1 [64] and the AAA+ chaperone CLPX.

In a generic chaperone mode, mitochondrial HSPs bind to exposed hydrophobic domains of unfolding proteins to establish a folding-competent state. This activity supports protein import into mitochondria under basal conditions [65] and is upregulated during organellar stress to reinforce proteostasis [66]. Substrate binding is typically low specificity, and intrinsically disordered regions in HSPs accommodate many geometries of interaction [67]. Crucially, the affinity and kinetics of HSP–substrate complexes are allosterically regulated by nucleotide binding: ADP-bound HSP70 variants form high-affinity complexes with slow exchange, whereas ATP binding accelerates association and dissociation, promoting substrate release [68] (Figure 2A). As a result, during energy crisis a negative cellular ATP economy shifts HSPs towards tighter, more durable substrate binding, functionally coupling metabolic stress to chaperone activity. This provides another example of how mitochondrial stress cascade, here combining thermal and metabolic cues, feeds directly into the proteostasis network.

Beyond these promiscuous substrate interactions, some HSPs also act in a more selective regulatory mode, binding defined “client” proteins to modulate their activity [69]. The mitochondrial HSP90-family chaperone TRAP1 (HSP75) exemplifies this behaviour: TRAP1 associates with respiratory chain complexes and can dampen mitochondrial respiration, thereby shifting metabolism towards aerobic glycolysis and reducing mitochondrial ROS production in several contexts [70] (Figure 2B). Together, these examples illustrate the duality of mitochondrial HSP-linked pathways: in a generic mode, HSPs preserve protein folding under stress, while in a regulatory mode, they participate in metabolic reprogramming that can either amplify or restrain mitochondrial ROS and proteostasis demand. Such HSP-mediated metabolic reprogramming of mitochondria is the consequence of selective interaction with key nodal proteins as follows.

Pyruvate dehydrogenase (PDH) is a key control point that determines whether pyruvate enters oxidative metabolism or is diverted toward anaerobic glycolysis. PDH activity oscillates between an active, dephosphorylated form and an inactive, phosphorylated form, with pyruvate dehydrogenase kinase (PDK) stimulated by acetyl-CoA and NADH and inhibited by CoA-SH and NAD^+^ [71]. TRAP1-mediated suppression of electron transport increases the NADH:NAD^+^ ratio [70] and promotes conditions that favour reverse electron transport at complex I [72], mimicking an energy-replete state that activates PDK and thereby inhibits PDH. Reverse electron transport in turn amplifies mitochondrial ROS production through partial reduction of oxygen both in mammalian cell models [42,72] and in Drosophila [73], which is particularly relevant because ROS directly inhibit pyruvate kinase M2 restricting metabolic flux into the TCA cycle (tricarboxylic acid or Krebs cycle) at this critical node [74]. Together, these interactions create a feed-forward loop in which TRAP1-dependent respiratory inhibition shunts carbon flux toward glycolysis while simultaneously elevating ROS (Figure 2C). Considering that such TRAP1-mediated reprogramming of mitochondria is a response to thermal stress, it is reasonable to question how amplified anaerobic glycolysis supports mitochondrial adaptation to this primary stressor.

A major consequence of enhanced glycolytic flux is accelerated regeneration of NAD^+^ via lactate dehydrogenase, which converts pyruvate to lactate [75] while oxidising NADH. This increased NAD^+^ availability is especially relevant for the NAD^+^-dependent deacetylase Sirt1, a master regulator of mitochondrial biogenesis and mitophagy [76] whose K_m_ for NAD^+^ lies within the physiological range [77]. Reduced NAD^+^ levels therefore limit Sirt1 activity [78], whereas elevated NAD^+^, occurring during high glycolytic flux, activates Sirt1, effectively allowing the enzyme to function as an NAD^+^ biosensor [77]. In the presence of sufficient NAD^+^, Sirt1 deacetylates heat shock factor-1 (HSF1), increasing its DNA-binding capacity and driving transcriptional upregulation of HSP70 [79,80], with important consequences for mitochondrial thermal quality control (Figure 2C).

Sirt1 simultaneously contributes to resolving ROS-related stress. Increased Sirt1 activity due to increased cellular NAD^+^ levels [81] promotes deacetylation and activation of PGC-1α [82], which enhances expression of manganese superoxide dismutase (MnSOD), and also upregulates MnSOD at the transcriptional level [83]. In parallel, mitochondrial Sirt3 complements Sirt1 by deacetylating and activating isocitrate dehydrogenase 2, boosting NADPH production [84] and supporting glutathione-dependent antioxidant defence. Thus, activation of glycolysis and sirtuin-mediated deacetylation primes mitochondria for a more oxidising environment by strengthening both chaperone capacity and antioxidant systems. This sequence illustrates our proposed cascade dynamic: adaptation to a primary thermal stressor engages TRAP1 with respiratory reprogramming generating ROS as a secondary stressor both in plants [85,86] and in mammalian models [87,88], which in turn amplifies Sirt1/Sirt3 signalling and HSF1-HSP responses to mitigate both the original thermal challenge and the ensuing oxidative stress.

Glyceraldehyde-3-phosphate dehydrogenase [89] and pyruvate kinase [74], two key rate-limiting enzymes in the distal segment of glycolysis, are directly inhibited by oxidative modification under ROS exposure [74,90]. The resulting decrease in lower glycolytic flux diverts glucose-6-phosphate into the oxidative phase of the pentose phosphate pathway (oxPPP), regenerating NADPH and replenishing antioxidant capacity, thereby enhancing cellular resilience in an oxidising milieu [91]. In parallel, ROS can enhance the activity of small GTPases such as Rheb [92], an allosteric activator of the mechanistic target of rapamycin complex 1 (mTORC1) [93]. This redox-mediated activation occurs via facilitated dissociation of GDP, likely through formation of unstable oxygenated nucleotide adducts that promote GDP release and GTP loading [94]; the detailed chemistry has been reviewed elsewhere [95]. Once activated, mTORC1 integrates inputs from cellular stressors to coordinate homeostatic responses [96]. In the context of thermal stress, relevant outputs include induction of heat shock proteins and other molecular chaperones: mTOR phosphorylates HSF1 on serine 326, a key residue for transcriptional activation [97], while HSF1, in turn, preserves mTORC1 integrity by antagonising JNK-dependent disassembly [98]. Activated HSF1 oligomerises, translocates to the nucleus, binds heat shock elements in HSP promoters, and upregulates HSP gene expression [98,99]. In addition, mTORC1 signalling stimulates proteasome biogenesis via Nrf1-dependent transcription of proteasome subunit genes [100], thereby preparing the cell for enhanced degradation of unfolded proteins.

Given the divergent outcomes of substrate-based versus client-based HSP interactions in mitochondria, it is natural to ask whether competition between substrates and clients helps determine how HSPs shape mitochondrial function under stress. Binding data offer an initial clue: for example, Hsp70 binds a model antigenic peptide with a dissociation constant in the low micromolar range [101], while Hsp72 binds the cochaperone DnaJA1 with a similarly micromolar affinity [102]. These comparable K_d_ values suggest that, in principle, the relative abundance of unfolded substrates versus regulatory clients could bias HSP engagement. Under conditions of high proteotoxic load, overwhelming substrate availability may transiently favour promiscuous substrate binding, directly supporting proteostasis while postponing client-mediated metabolic reprogramming. We therefore propose that substrate–client competition at HSPs could help set the timing and extent of mitochondrial metabolic rewiring, including shunting of carbon into the pentose phosphate pathway in anticipation of increased ROS production during thermal stress [86].

### 2.3. Oxidation of Mitochondrial Proteins

Oxidation of mitochondrial proteins can be a physiological, highly localised process, as in the activity of the Mia40–Erv1 disulfide relay, which introduces disulfide bonds into imported intermembrane space (IMS) proteins to stabilise the folded state [103,104,105]. In contrast, when the mitochondrial environment shifts towards a more oxidising milieu—for example, due to electron leakage from the respiratory chain [15,16], ROS can induce broad, non-specific oxidation of proteins throughout the organelle [106] (Table 1). A detailed account of ROS generation sites and mechanisms is available elsewhere [15], so here we summarise key biophysical principles to support a systems-level view of how mitochondrial quality control modules detect, buffer, and repair oxidative damage.

During normal respiration, mitochondria generate ROS as electrons leak from the electron transport chain (ETC) to molecular oxygen, producing short-lived superoxide O_2_^•−^ [18,127]. Complex I primarily releases superoxide anion into the matrix, whereas complex III releases superoxide anion into both the matrix and the IMS [15]. ROS production is further amplified when NADH accumulates as the ETC stalls. Under these conditions, dihydroxyacetone phosphate can be reduced to glycerol-3-phosphate to regenerate NAD^+^ via mitochondrial glycerol-3-phosphate dehydrogenase, which transfers electrons to ubiquinone, generating substantial amounts of superoxide anion as a byproduct [128]. Superoxide anion is then dismutated by manganese superoxide dismutase (MnSOD) to hydrogen peroxide (H_2_O_2_) [129], consuming two superoxide radicals and two protons to yield H_2_O_2_ and O_2_. H_2_O_2_ can be detoxified further by glutathione peroxidase, which uses reduced glutathione (GSH) to convert H_2_O_2_ into water (and glutathione disulfide) [130], or, in the presence of ferrous iron, participate in Fenton chemistry to generate highly reactive hydroxyl radicals (^•^OH) [131]. These distinct ROS differ markedly in half-life, diffusion range, and reactivity, which in turn shapes the spatial and temporal deployment of mitochondrial QC mechanisms discussed in the following sections.

Before discussing QC mechanisms engaged by protein oxidation, it is useful to distinguish oxidative modifications that support adaptation to ROS from those that represent detrimental damage. Oxidation of the adenine nucleotide translocator (ANT) exemplifies a modification that may facilitate adaptation to oxidative stress [116,117,121]. Oxidative carbonylation of ANT decreases the ADP^3−^/ATP^4−^ exchange capacity [121], reducing electrogenic export of negative charge and favouring accumulation of ATP^4−^ in the matrix. Because each exchange cycle normally exports one net negative charge, slowing this flux is expected to promote mitochondrial hyperpolarisation (net gain of 1e^−^ per retained ATP^4−^ molecule in the matrix) [38,132]:

$${ADP}^{3-}\overset{{ANT}^{off}}{\to }cytosol\;\;\;\;\;\;\;\;{ATP}^{4-}\overset{{ANT}^{off}}{\to }matrix\;\;\overset{Net\;gain}{\Rightarrow }\;1\left[e^{-}\right]\;gain\;in\;matrix/{ATP}^{4-}$$

In an Ant^off^ state (i.e., when ADP^3−^/ATP^4−^ exchange is repressed), reversal of translocase activity promotes accumulation of ATP^4−^ in the matrix and ADP^3−^ in the cytosol. Consequently, each abolished exchange cycle results in a net gain of one negative charge across the inner mitochondrial membrane.

**Figure 3:**
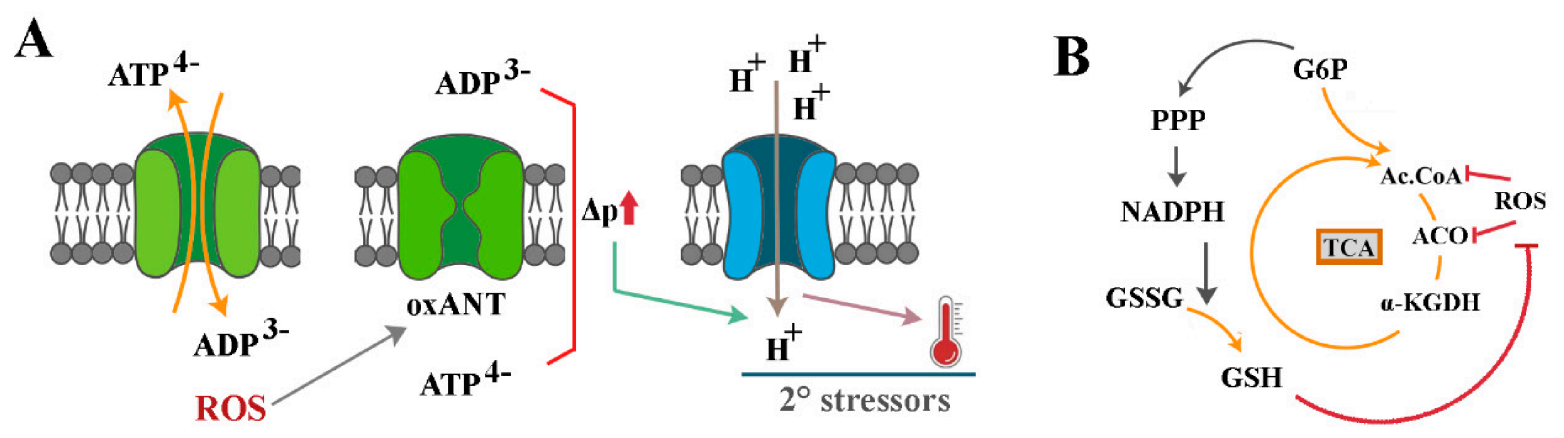
ROS-dependent modulation of adenine nucleotide transport and redox buffering in mitochondrial stress cascades. (**A**) Regulation of the adenine nucleotide translocator (ANT) and proton flux by ROS. Under basal conditions, ANT exchanges ATP^4−^ and ADP^3−^ across the inner mitochondrial membrane to support ATP export. ROS-mediated oxidation of ANT (oxANT) alters its transport properties, favouring a flux pattern that contributes to mitochondrial hyperpolarisation (Δp↑). Elevated Δp, in turn, increases proton leak through dedicated proton-conducting pathways, converting part of the primary electrochemical stress into secondary stressors such as heat and altered proton flux. (**B**) Integration of the pentose phosphate pathway (PPP), glutathione cycle and tricarboxylic acid (TCA) cycle in ROS handling. Glucose-6-phosphate (G6P) enters the PPP to generate NADPH, which maintains glutathione in its reduced form (GSH) from oxidised glutathione (GSSG). Within the TCA cycle, enzymes such as aconitase (ACO) and α-ketoglutarate dehydrogenase (α-KGDH) are both sources and targets of ROS, linking redox stress to flux through acetyl-CoA and intermediate metabolites. Together, these pathways form a coupled network in which ROS not only signify mitochondrial stress but also dynamically reshape nucleotide transport, membrane potential and redox buffering capacity.

Under physiological conditions, ANT catalyses ~1500–2000 exchanges per minute and accounts for roughly 10% of inner membrane protein in rat liver mitochondria [133]. Accordingly, this activity measurably lowers ΔΨ by ~10–20 mV in state 3 [134] (Figure 3A). When ANT is oxidatively impaired, the resulting hyperpolarisation recruits oligomycin-insensitive proton leak pathways [134] that act to prevent excessive ΔΨ and thereby limit further ROS production. Such non-ohmic proton leak converts a portion of the proton motive force into heat [135], contributing to thermogenesis, while elevated ΔΨ in turn increases proton conductance through uncoupling proteins once the inhibitory influence of matrix ATP [136] is overcome [137]. In this way, a primary ROS insult is partially transformed into second-tier stressors, namely matrix acidification and increased temperature, that engage thermal adaptation and the matrix re-alkalinisation mechanisms described earlier (Figure 3A).

Oxidation of other mitochondrial enzymes can also be functionally adaptive. Aconitase and α-ketoglutarate dehydrogenase are particularly sensitive to H_2_O_2_. Inactivation of enzymatic activity under mild oxidative stress limits TCA flux and favours re-routing of carbon toward the pentose phosphate pathway [138,139] (Figure 3B), thereby supporting NADPH regeneration and antioxidant defence. Similarly, disulfide-mediated mitofusin oligomerisation under oxidising conditions promotes mitochondrial fusion [126], which facilitates complementation among partially damaged mitochondria and creates shared QC hubs that dilute damage and enhance repair capacity [140]. In contrast, oxidation of manganese superoxide dismutase diminishes catalytic activity and compromises detoxification of superoxide anion [108], clearly representing a maladaptive modification that exacerbates oxidative injury.

Collectively, these examples suggest that “adaptive” oxidation events either help dissipate the primary ROS stress directly (for example, by promoting mild uncoupling) or convert it into secondary stress modalities, such as heat, that can be managed by additional QC systems without compromising core mitochondrial function. Importantly, adaptive oxidation is not restricted to mitochondrial proteins. Redox-sensitive disulfide bond formation in cytosolic and nuclear factors can indirectly support mitochondrial QC. For instance, intermolecular disulfide bond formation between specific cysteines in heat shock factor 1 (HSF1) drives trimerisation, DNA binding, and nuclear accumulation under stress, enhancing transcription of nuclear-encoded HSP genes that contribute to mitochondrial proteostasis [141].

Oxidative protein modifications span a spectrum from fully reversible regulatory events to irreversible damage that necessitates degradation [142]. For sulfur-containing amino acids, cysteine sulfenic (–SOH) and sulfinic acid (–SO_2_H) modifications can often be reversed by glutaredoxin and sulfiredoxin, either directly or via cycles of S-glutathionylation and deglutathionylation. In contrast, cysteine sulfonic acid (–SO_3_H) is generally irreversible and targets proteins for proteasomal degradation. Methionine sulfoxide can be reduced back to methionine by methionine sulfoxide reductases [143], whereas methionine sulfone is essentially refractory to further modification. Protein carbonylation, characterised by the introduction of reactive aldehyde or ketone groups into side chains, is likewise considered a largely irreversible lesion associated with loss of function marking proteins for turnover [144]. As discussed in subsequent sections, mitochondrial QC pathways are organised to exploit reversible oxidative modifications for signalling and adaptation, while identifying and clearing proteins that have crossed the threshold into irreversible damage.

The coexistence of reversible regulatory oxidations and irreversible damaging lesions, distributed across a broad spectrum of mitochondrial targets, raises an unresolved question: at what point do adaptive responses become a metabolic burden that warrants elimination of the organelle rather than continued repair? Oxidative modifications that promote mild uncoupling, re-route flux into the pentose phosphate pathway, or drive fusion and HSP induction can clearly be beneficial in the short term, but each comes at a cost in ATP, reducing equivalents, and biosynthetic capacity. As damage accumulates, the energetic and biosynthetic resources required to sustain proteostasis, antioxidant defence and membrane potential may eventually exceed the output capacity of the compromised mitochondrion.

From a systems perspective, mitochondria must therefore operate with implicit “decision thresholds” that distinguish salvageable from terminally damaged states. Below these thresholds, reversible cysteine and methionine oxidations, transient carbonylation of selected enzymes, and redox-driven changes in dynamics or transport can be reversed or compensated for, allowing the organelle to re-enter productive bioenergetic states. Beyond them, however, the accumulation of irreversible lesions such as extensive protein carbonylation, sulfonylation, and sulfone formation, or persistent inactivation of key nodes like ANT, TCA enzymes, and SODs progressively locks the system into a low-output, high-maintenance configuration. In this regime, continued deployment of adaptive loops (e.g., sustained uncoupling, chronic PPP activation, maximal chaperone and protease activity) increasingly drains cellular ATP and reducing power without restoring adequate mitochondrial function.

It is likely that cells integrate multiple signals to identify when this tipping point has been reached, including sustained loss of membrane potential, stalled respiration despite maximal substrate availability, chronic activation of stress-response transcription factors and accumulation of specific damage markers in the matrix and inner membrane. Once such criteria are met, the balance is expected to shift from costly repair toward selective removal of the organelle via mitophagy and related quality control pathways. In this view, the same oxidative and metabolic changes that initially facilitate adaptation to stress gradually serve as readouts of an organelle that has exhausted reparative capacity, marking the transition from a salvage-oriented to an elimination-oriented QC regime.

### 2.4. Mitochondrial Protein Glycosylation

Protein glycosylation is a fundamental physiological process that shapes protein folding, trafficking, and stability. In the endoplasmic reticulum (ER), nascent polypeptides acquire N-linked glycans that promote proper folding [145] by engaging lectin-type chaperones such as calnexin and calreticulin [146]. These glycans are subsequently trimmed and remodelled during passage through the Golgi apparatus as part of glycoprotein maturation [147]. In addition to this constitutive role, glycosylation can participate directly in adaptation to acute stress [148], through the attachment of glycans to asparagine (N-glycosylation), serine/threonine (O-glycosylation, including O-GlcNAc), or, more rarely, tryptophan (C-glycosylation) [149]. In mitochondrial biology and pathology, emerging evidence suggests that glycosylation plays a role by modulating the activity of mitochondrial proteins [150]. In neuroinflammation, the appearance of N- and O-linked glycans appears to coincide with the transition from acute to chronic inflammation [151]. Recent findings have also hinted at an enrichment of glycosylated mitochondrial proteins in Parkinson’s disease, potentially contributing to increased oxidative stress in the disease [152]. In the discussion that follows, we first consider the broader role of glycosylation in stress adaptation and then examine glycan-mediated modulation of mitochondrial activity.

Protein glycosylation interfaces with stress responses at multiple levels. Acute heat stress induces “prompt” N-glycosylation of specific plasma-membrane proteins within minutes [153], even as basal glycosylation of other proteins is suppressed. Pharmacological elevation of O-GlcNAc levels increases the expression of HSP72 and HSP40 during thermal stress [148], and mechanistic studies suggest that O-GlcNAcylation restrains glycogen synthase kinase-3β (GSK3β), thereby reducing inhibitory Ser303 phosphorylation of HSF1 and sustaining its transcriptional activity [154]. Beyond regulating chaperone expression, N-glycosylation can directly reduce protein aggregation: glycans positioned near aggregation-prone regions sterically hinder self-assembly, and loss of such glycans promotes aggregation in cells [155]. Thus, stress-induced glycosylation supports proteostasis both by enhancing HSP induction and by physically stabilising vulnerable proteins.

Stress-responsive glycosylation also modulates broader proteostasis networks. The ER-resident transcription factor Nrf1/NFE2L1 senses proteasome dysfunction and mediates a “bounce-back” response that upregulates proteasome subunit genes [156,157]. For Nrf1 to become fully functional, it must undergo a cycle of N-linked glycosylation in the ER lumen followed by retro-translocation and cytosolic deglycosylation, during which specific N-glycosylated asparagine residues are edited to aspartic acid [158]. This glycosylation–deglycosylation cycle is therefore integral to activation of a key QC transcription factor and to the adaptive expansion of proteasome capacity. In the context of mitochondrial stress, such glycosylation-dependent signalling pathways indirectly support mitochondrial quality control by enhancing cellular capacity to degrade oxidised and misfolded mitochondrial proteins.

Stress-induced O- and N-glycosylation of multiple mitochondrial inner and outer membrane proteins has been documented (Table 2), but for most targets the functional consequences remain incompletely defined. For example, augmenting cardiac O-GlcNAc levels reduces mitochondrial Ca^2+^ overload and ROS generation during reperfusion after ischemia, consistent with a protective effect on mitochondrial permeability transition, yet the underlying molecular mechanisms are not resolved [159]. In another study, elevated O-GlcNAcylation of mitochondrial complex I, complex IV, VDAC, and SERCA correlated with reduced oxidative capacity in hearts from low-capacity rats after exercise [160], but whether altered Ca^2+^ handling via glycosylated VDAC contributed to this phenotype was not examined. Beyond these functional hints, the presence of organelle-localised glycosylation machinery is supported by evidence for a mitochondrial splice isoform of O-GlcNAc transferase (mOGT) that contains a targeting sequence for association with the inner membrane [161], as well as reports of mitochondrial N-linked glycoconjugates [162]. Taken together, these findings suggest that glyco-conjugation is likely to modulate mitochondrial responses to stress, but the scope and mechanisms of this regulation remain largely unexplored.

At a broader regulatory level, oxidative stress and O-GlcNAcylation are tightly intertwined. Acute H_2_O_2_ exposure increases O-GlcNAcylation of proteins involved in chromatin remodelling, transcriptional control, and RNA/stress-granule biology [167,168], in part because upregulated fatty acid synthase binds to and inhibits a pool of O-GlcNAcase (OGA), the enzyme that removes O-GlcNAc from proteins [169]. Among these targets, O-GlcNAcylation of Sirt1 at Ser549 is particularly relevant for mitochondrial quality control: this modification directly enhances Sirt1 deacetylase activity and promotes cyto-protection under genotoxic, oxidative, and metabolic stress [170]. As noted above, increased Sirt1 activity contributes to ROS neutralisation via PGC-1α activation [82] and MnSOD induction [83,171], thereby strengthening mitochondrial antioxidant defences.

Convergence of O-GlcNAc signalling and redox regulation is evident in central carbon metabolism as well. Under hypoxia, O-GlcNAcylation of phosphofructokinase-1 at Ser529 suppresses activity and diverts glucose flux away from glycolysis toward the oxidative branch of the pentose phosphate pathway (oxPPP), increasing NADPH and reduced glutathione levels [172]. In parallel, O-GlcNAcylation of glucose-6-phosphate dehydrogenase, the rate-limiting enzyme of the pentose phosphate pathway, enhances activity and further boosts oxPPP-dependent production of reducing equivalents for antioxidant defence [173].

The antioxidant transcription factor Nrf2 provides another point of intersection between the hexosamine biosynthetic pathway and mitochondrial QC [174]. Nrf2 target genes play key roles in mitochondrial antioxidant and detoxification systems [175], and Nrf2 stability is controlled by Keap1–Cul3-dependent ubiquitination and proteasomal degradation. O-GlcNAcylation of Keap1 at Ser104 is required for efficient formation of this complex and thus for Nrf2 turnover [176], linking UDP-GlcNAc availability [177] to Nrf2 signalling [178]. When glucose and other hexosamine pathway inputs are abundant, enhanced O-GlcNAcylation of Keap1 promotes Nrf2 degradation [179], whereas glucose deprivation reduces Keap1 glycosylation, stabilises Nrf2, and activates Nrf2-dependent antioxidant programmes. Nrf2 activation, in turn, increases mitochondrial fatty acid β-oxidation [178] and upregulates multiple antioxidant enzymes, further adjusting mitochondrial redox capacity [180]. In this way, O-GlcNAc acts as a nutrient- and stress-sensitive modifier that tunes both mitochondrial function and the surrounding QC network.

Viewed through the lens of cascade dynamics, stress- and nutrient-regulated glycosylation represents an additional tier at which primary mitochondrial stress signals are integrated and transformed into downstream adaptive programmes. By tuning the activities of Sirt1, key glycolytic and pentose phosphate enzymes, together with redox-related transcription factors such as Nrf2, O- and N-linked glycosylation, help convert mitochondrial ROS and metabolic imbalance into coordinated adjustments of antioxidant capacity, proteasome function, and chaperone induction, thereby extending the effective range of mitochondrial quality control without directly perturbing core bioenergetic machinery. In this framework, primary mitochondrial stressors (thermal and oxidative) engage glycosylation-dependent signalling as secondary and tertiary stress nodes, which in turn redistribute the burden of adaptation onto cytosolic and nuclear QC modules while preserving essential mitochondrial output.

### 2.5. Mitochondrial ATP-Dependent Proteases

Mitochondrial proteases have been reviewed extensively elsewhere [181,182,183]. Beyond the processing peptidases that remove targeting sequences, a core set of enzymes play central roles in protein quality control: the matrix proteases LonP1 and ClpP, the inner-membrane AAA+ proteases (m-AAA and i-AAA), and the IMS-resident proteases Atp23 and Htra2 [182,184]. LonP1 and ClpP are soluble in the matrix, whereas m-AAA is anchored in the inner membrane with the catalytic site facing the matrix while i-AAA (YME1L) faces the IMS. Atp23 and Htra2/Omi reside in the IMS. By virtue of the membrane localisation, AAA proteases continuously surveil oxidative phosphorylation (OXPHOS) complexes [185,186,187], degrading damaged subunits and, in some cases, promoting reassembly of respiratory chain complexes via chaperone-like activities [188].

Lon protease recognises substrates by sensing exposed hydrophobic regions within otherwise soluble proteins [189] and uses ATP hydrolysis to unfold and degrade these targets. ADP is a potent competitive inhibitor of Lon, with 10–20-fold higher affinity than ATP in the absence of substrate [190]; substrate binding decreases ADP affinity by roughly one order of magnitude, thereby promoting ADP/ATP exchange and sustaining proteolysis even under fluctuating energy conditions [190]. In addition to misfolded proteins, Lon rapidly degrades oxidised mitochondrial proteins [43] and is transcriptionally induced by hypoxia-inducible factor-1 (HIF-1) [191]. Under hypoxia, HIF-1 upregulates both LONP1 and the COX4-2 isoform of cytochrome c oxidase while Lon selectively degrades the COX4-1 isoform, enabling a COX4-1→COX4-2 switch that optimises electron transfer, improves ATP yield, and reduces ROS production at low oxygen tension [191]. This proteolytic rewiring of complex IV becomes particularly significant given that mitochondrial ROS stabilise HIF-1α by inhibiting prolyl hydroxylases and can also enhance HIF-1α transcription [192], permitting accumulation of HIF-1 in the absence of profound hypoxia [193,194]. From this perspective, ROS-mediated activation of HIF-1 amplifies Lon expression and activity, priming mitochondria for accelerated clearance of oxidised proteins and dynamic adjustment of ETC composition.

The matrix ClpXP protease complex similarly contributes to mitochondrial quality control by degrading denatured or misfolded proteins [195], but it also plays a specialised signalling role in the mitochondrial unfolded protein response (UPRmt), particularly in *C. elegans*. Under conditions where stress-induced misfolded proteins accumulate and exceed chaperone capacity in the matrix [196], ClpP degrades these substrates into peptides that are exported to the cytosol via the HAF-1 ABC transporter [197]. Peptide efflux impairs further protein import through the TOM/TIM complexes, allowing the bZIP transcription factor ATFS-1—which carries both an N-terminal mitochondrial targeting sequence and a nuclear localisation sequence—to escape mitochondrial import, accumulate in the nucleus, and activate a protective transcriptional programme characterised by induction of mitochondrial chaperones and proteases [198]. In this way, matrix protease activity is re-purposed as a retrograde signal, converting an excess of unfolded mitochondrial proteins into a nuclear gene-expression response that expands the organelle’s QC capacity.

Together, these protease circuits help define the boundary between repairable and terminally damaged mitochondria. While LonP1, ClpXP, and the AAA proteases initially act to restore function by selectively degrading oxidised or misfolded proteins and rewiring ETC composition, persistent activation of these pathways, particularly when accompanied by sustained loss of membrane potential and chronic UPRmt signalling, likely serves as a readout that the energetic cost of repair exceeds the bioenergetic output of the organelle. At this point, the same cues that first engaged local proteolysis are expected to converge with mitophagy pathways, shifting the QC regime from salvage of individual components to elimination of the entire mitochondrion.

### 2.6. Mitochondrial Antioxidant and Redox Control Systems

Mitochondria are equipped with multiple redox buffers and antioxidants (Table 3). A major enzymatic defence is manganese superoxide dismutase (MnSOD/SOD2), which catalyses the dismutation of superoxide anion to H_2_O_2_. The importance of MnSOD becomes clear when comparing the kinetics of superoxide production and decay: although spontaneous dismutation at near-neutral pH is relatively fast (k ≈ 8.5 × 10^7^ M^−1^ s^−1^), this rate is still well below typical rates of superoxide generation in mitochondria (k up to ~2 × 10^10^ M^−1^ s^−1^) [199]. MnSOD accelerates dismutation toward diffusion-controlled values (k ≈ 10^8^–10^9^ M^−1^ s^−1^) [199], thereby preventing accumulation of superoxide. Loss of MnSOD activity leads to rapid oxidation and inactivation of redox-sensitive Krebs-cycle enzymes such as aconitase and succinate dehydrogenase, underscoring a central role for MnSOD in maintaining matrix redox poise [200,201]. However, MnSOD activity must be maintained within an optimal window. Insufficient activity leaves cells vulnerable to superoxide-mediated damage [200], whereas excessive MnSOD expression, particularly in the absence of matching H_2_O_2_-scavenging capacity, can increase H_2_O_2_ levels and unmask peroxidase activity that promotes oxidation of other mitochondrial substrates [202,203,204]. Multiple signalling pathways therefore converge to fine-tune MnSOD [205]. At the post-translational level, Sirt3-dependent deacetylation activates MnSOD [206,207,208,209], while parallel Sirt3-mediated deacetylation stabilises FOXO3 and promotes nuclear translocation of the entity, driving transcriptional upregulation of MnSOD and catalase [210]. Nitration of MnSOD diminishes catalytic activity [211], but de-nitration can restore function [212]; hypoxia-induced de-nitration has been proposed to precondition mitochondria for subsequent re-oxygenation and ROS bursts [212]. In addition, NF-κB and related stress-responsive transcription factors trans-activate the MnSOD promoter [213,214], aligning MnSOD induction with broader inflammatory and oxidative stress programmes [215,216]. Collectively, these regulatory layers allow MnSOD activity to track both current and anticipated mitochondrial ROS loads, with H_2_O_2_ emerging as the principal product that must then be processed by downstream antioxidant systems.

The tripeptide glutathione (γ-L-glutamyl-L-cysteinyl-glycine, GSH) constitutes the dominant small-molecule antioxidant in the mitochondrial matrix, where it reduces H_2_O_2_ to water via glutathione peroxidases. In this cycle, GSH is oxidised to GSSG and then regenerated by NADPH-dependent glutathione reductase [130]. In parallel, the glutaredoxin system, supported by GSH and NADPH, reduces protein disulfides and protein–glutathione mixed disulfides [217], while the thioredoxin system uses thioredoxin reductase and NADPH to maintain protein thiols in a reduced state [218]. In all cases, sustained antioxidant capacity depends on adequate NADPH availability.

To supply mitochondrial NADPH, Sirt3 deacetylates and activates isocitrate dehydrogenase 2 (IDH2) [219], increasing NADPH production in the matrix and thereby supporting regeneration of reduced glutathione and other thiol-based antioxidant systems [220]. This Sirt3-IDH2 axis parallels Sirt3’s activation of MnSOD, helping to ensure that accelerated dismutation of superoxide is matched by sufficient H_2_O_2_-reducing capacity. When mitochondrial ROS production outstrips the NADPH-generating capacity of the Krebs cycle and associated dehydrogenases, cells recruit cytosolic pathways to bolster NADPH supply. Under such conditions, oxidative inhibition [74,90] of glyceraldehyde-3-phosphate dehydrogenase [89] and pyruvate kinase [74] diverts glucose-derived carbon from lower glycolysis into the oxidative arm of the pentose phosphate pathway, expanding the cytosolic NADPH pool and enhancing the ability to regenerate GSH and other antioxidants [91]. This hierarchical mobilisation of mitochondrial and cytosolic NADPH sources exemplifies how redox stress at the organellar level propagates through metabolic networks to reinforce mitochondrial quality control.

**Table 3:** An overview of mitochondrial antioxidants.

Antioxidant System	Key Redox Couple	E°’ (mV)	Concentration	Ref.
SOD2 (MnSOD)	O_2_^−^/H_2_O_2_	+320 to +440	~10 μM	[221]
Glutathione (GSH)	GSSG/GSH	−240	~5 mM	[222,223,224]
Thioredoxin (Trx2)	Trx-(SH)_2_/Trx-S_2_	−270	~10–50 μM	[225]
Peroxiredoxin 3	Prx3-SOH/Prx3-SH	~290	~20–50 μM	[226]
Ubiquinol (CoQ10)	Q/QH_2_	+60 to +100	~1–5 μM	[227]
α-Tocopherol	Toc/TocH	+480 to +500	~0.1–1 μM	[228]

Framed in terms of cascade dynamics, mitochondrial antioxidant systems illustrate how a primary stress signal is progressively redistributed across metabolic layers. Superoxide generated at the respiratory chain is first transformed into H_2_O_2_ by MnSOD; this, in turn, recruits GSH and thioredoxin systems whose capacity is constrained by matrix NADPH, prompting Sirt3-dependent activation of IDH2 and, under higher stress, glycolytic re-routing into the pentose phosphate pathway to expand cytosolic NADPH. Each step partially dissipates the original ROS insult while shifting part of the adaptive burden from the mitochondrion to the surrounding cellular metabolic network, delaying the point at which damage becomes irreparable, and organelle elimination is favoured over repair.

### 2.7. Quality Control of Mitochondrial Lipids

Beyond a structural role, mitochondrial lipids interact directly with components of the electron transport chain to modulate activity [229,230,231]. These lipids are particularly vulnerable to peroxidation because of proximity to ROS-generating respiratory complexes, creating a need for quality control mechanisms that restore the non-oxidised membrane state. Before considering how damaged lipids are repaired, it is useful to examine how controlled lipid oxidation can itself contribute to adaptation in an organelle that is a major source of oxidants [15].

Oxidised cardiolipin (oxCL) is hydrolysed by the inner-membrane enzyme iPLA_2_γ (PNPLA8) to generate monolysocardiolipin (MLCL) [232], which is then re-acylated by Tafazzin using linoleoyl chains from phosphatidylcholine to regenerate mature tetralinoleoyl-cardiolipin [233]. This de-acylation–re-acylation cycle removes peroxidised acyl chains and maintains the characteristic cardiolipin composition required for optimal respiratory super-complex function. oxCL species adopt disordered, non-lamellar conformations that increase access of the sn-2 acyl chains to the active site serine of iPLA_2_γ, thereby selectively targeting damaged lipids for turnover [232]. In addition, iPLA_2_γ can be directly activated by oxidants in reconstituted systems [234]. This activity is further enhanced by MAPK signalling, as MEK1-mediated phosphorylation of non-catalytic serines increases catalytic efficiency [235]. Because ROS also promote Ras/MAPK activation via redox-facilitated GDP release and GTP loading [94], mitochondrial lipid QC is regulated at multiple levels: oxidised acyl chains become preferred substrates, and the same ROS that produce oxCL can directly and indirectly stimulate iPLA_2_γ activity.

The consequences of iPLA_2_γ activation extend beyond cardiolipin repair. Free fatty acids liberated by iPLA_2_γ associate with UCP2 in pancreatic β-cells [236] and with ANT1 in other tissues, promoting FA-dependent proton leak across the inner membrane. This controlled uncoupling partially collapses the proton motive force (particularly ΔpH), thereby attenuating mitochondrial superoxide production [236], especially under conditions that favour reverse electron transport (RET) at complex I. Thermodynamically, forward electron transfer from NADH to ubiquinone is favoured when the redox potential difference between the NAD^+^/NADH and CoQ/CoQH_2_ couples provides sufficient free energy to pump four protons against Δp [237]; at high Δp, this inequality is no longer satisfied and RET with robust ROS generation becomes more likely. By dissipating Δp through FA-dependent proton conductance via UCP2 and ANT, iPLA_2_γ-released fatty acids shift the system back toward forward electron flow and reduce RET-mediated ROS.

ANT’s dual role as an ADP/ATP exchanger and FA-dependent proton carrier further links lipid signalling to nucleotide and Δp homeostasis [238,239,240]. Long-chain fatty acids enable ANT1-mediated H^+^ transport that is inhibited by purine nucleotides, while dissipation of Δp [238,239,240] reduces the driving force for ADP^3−^ import [241], relieving nucleotide-dependent inhibition of UCPs and amplifying proton leak. The resulting drop in matrix pH engages reverse-mode ATP synthase, which exports protons to re-alkalinise the matrix at the cost of ATP hydrolysis, leading to ADP^3−^ accumulation [31]. Elevated matrix ADP^3−^ not only restores physiological adenine nucleotide ratios and restrains UCP activity but also drives ATP^4−^/ADP^3−^ exchange via ANT, whose electrogenic transport contributes to repolarisation of the inner membrane. In parallel, increased proton leak enhances mitochondrial thermogenesis, which, as discussed earlier, can propagate to the nucleus to trigger HSF1 activation and heat-shock responses that expand the mitochondrial QC network.

Taken together, these observations illustrate how mitochondria adapt to ROS by sequentially converting a primary oxidative stress into secondary and tertiary perturbations of lipids, Δp, and pH. Oxidised cardiolipin activates iPLA_2_γ-dependent cardiolipin remodelling and FA release; FA-driven uncoupling via UCP2 and ANT lowers Δp and curtails RET-mediated ROS. The resulting matrix acidification and thermogenesis recruit ATP synthase, adenine nucleotide exchange, and HSP-mediated QC to re-establish homeostasis. This lipid-centred cascade represents yet another instance of the broader stress-conversion dynamics that underpin mitochondrial quality control.

### 2.8. Mitochondrial DNA: A Sensor and Mitigator of Organellar Stress

Nucleic acids can act as sensors of environmental stress by virtue of a polymeric structure and the capacity to undergo conformation changes. For instance, the abundance of many cytosolic HSPs depends on cryptic thermo-sensitive elements in 5′-untranslated regions that modulate ribosome access [242,243]. Bacterial “RNA thermometers” [244] such as the ROSE element provide a classic example: this multi-hairpin structure masks the Shine–Dalgarno sequence at low temperature [245,246], and gradual melting between ~30 and 42 °C exposes the ribosome-binding site to permit translation of heat-shock mRNAs [247].

Recent work suggests that a related principle may operate in the mitochondrial genome, where structural elements in DNA rather than RNA could act as cryptic thermosensors during neuronal differentiation [248]. While these findings remain preliminary and require validation across broader model systems, we provide an overview of this emerging model here. In the strand-displacement model of mtDNA replication [249,250], leading-strand (H-strand) synthesis initiates at O_H_ [251], and lagging-strand (L-strand) synthesis begins only after ~11 kb of H-strand have been synthesised [252], when the origin of light-strand replication (OriL) becomes single-stranded and folds into a short stem–loop that serves as the primer-binding site for L-strand synthesis. Under physiological conditions, this hairpin licences L-strand replication [253]. However, elevated mitochondrial temperature could destabilise the OriL stem–loop, repressing L-strand initiation and generating a “quasi-replication” mode in which only the parental light strand is copied [248]. In this scenario, the displaced heavy strand remains single-stranded for extended periods. Although mitochondrial single-stranded DNA-binding protein (mtSSBP1) normally coats and protects nascent single-stranded DNA [254], mtSSBP1 is thermosensitive and the DNA binding can be disrupted by heat [248]. Aside from this direct impact, heat-induced HSF-1 suppresses single-stranded DNA-binding protein 1 (SSBP1) oligomer formation [255], critical for binding to DNA and regulation of mtDNA replication [256]. In consequence, exposed H-strand segments function as natural antisense templates that base-pair with complementary heavy-strand mRNAs, forming DNA–RNA hybrids that are subsequently processed by RNase H1 [248]. Degradation of these hybrids would reduce mitochondrial transcript levels, dampen ETC assembly, and thereby limit further ROS and heat production [248]. In this view, heat-triggered changes in OriL dynamics and mtSSBP1 function could convert mtDNA from a passive template into an active thermal sensor that feeds back on mitochondrial output. However, the extent to which this thermosensory function operates broadly in different cell types and organ systems remains to be established.

In addition to acting as structural thermosensors, mitochondrial genomes are highly susceptible to oxidative damage [257]. Reactive oxygen species can generate more than 20 different DNA base adducts [258], and mtDNA from hepatocytes often harbours several-fold higher levels of 8-oxo-deoxyguanosine than nuclear DNA [259]. Mitochondria repair oxidised bases primarily via base excision repair (BER) [260], in which DNA glycosylases recognise and excise damaged bases, creating abasic sites that are processed by AP endonucleases, followed by gap filling by DNA polymerase γ and ligation by DNA ligase III [261]. In mitochondria, glycosylases that remove oxidised DNA bases possess an associated lyase activity whereby the DNA backbone is nicked 3′ to the lesion after removal of the damaged base [262]. The apurinic site generated by DNA glycolyase is further processed by either APE1 or PNK to render the free ends suitable for the gap-filling polymerase, the mitochondrial Polγ [263]. Ultimately, the repair of the damaged site is completed by catalytic activity of DNA ligase III that seals the gap [264]. Many glycosylases that act in mitochondria are encoded by the same genes as their nuclear counterparts but are targeted to the organelle through alternative transcription start sites or splicing that generate N-terminal mitochondrial targeting sequences [265,266]. Efficient BER is therefore a key element of mtDNA quality control; failure to repair oxidative lesions can compromise replication and transcription and ultimately promote mitophagy.

Nuclear DNA damage also indirectly constrains mitochondrial QC. Because mitochondrial proteins are almost entirely nuclear-encoded, oxidative lesions in nuclear DNA can impair transcriptional replenishment of mitochondrial QC factors. Moreover, activation of poly(ADP-ribose) polymerase-1 (PARP1) at nuclear damage sites consumes large amounts of NAD^+^, as PARP1 uses NAD^+^ to build poly(ADP-ribose) chains on the entity and nearby chromatin proteins [267]. The PARylation of DNA damage sites contributes to more than 90% of total PARylation events in a cell [268,269]. PARPs use NAD^+^ to attach long chains of ADP-ribose (PAR) onto the enzyme (auto-PARylation) and to nearby chromatin proteins (e.g., histones) [270]. In response to severe DNA damage, PARP-dependent PARylation can account for the majority of cellular PAR and deplete 80–90% of the NAD^+^ pool [271]. This depletion triggers a cell-wide metabolic shift toward oxidative phosphorylation to regenerate NAD^+^ [271] and can impair NAD^+^-dependent deacetylases such as Sirt1, which are important for mitochondrial biogenesis, antioxidant defence, and UPRmt signalling. Conversely, PARP1 inhibition increases NAD^+^ levels and Sirt1 activity in tissues such as brown adipose, enhancing mitochondrial function [272]. Thus, while mtDNA itself may act as a thermal and oxidative sensor that modulates mitochondrial activity, both mitochondrial and nuclear DNA damage require dedicated QC pathways whose operation is tightly linked to the same NAD^+^ pool that powers key mitochondrial adaptive responses.

## 3. Mitochondrial Quality Control at Sub-Organellar and Organellar Level

Mitochondria-derived vesicles (MDVs) are small vesicular carriers that bud from mitochondria to deliver selected cargo to endosomes and lysosomes [273], thereby removing damaged components without sacrificing the entire organelle. MDV formation occurs at low levels under basal conditions but is rapidly upregulated, within minutes to a few hours, after exposure to mitochondrial stressors [274], and complements other QC pathways by selectively routing [275] oxidised or irreversibly damaged proteins and lipids to lysosomal compartments [274,276]. Stress-induced MDV biogenesis frequently depends on the PINK1–Parkin pathway [274]. In healthy, polarised mitochondria, PINK1 is imported through the TOM complex into the inner membrane, where the intramembrane protease PARL cleaves it to expose an N-terminal destabilising residue [277]; the truncated PINK1 is then retro-translocated and degraded via the N-end rule pathway [277], preventing signalling. Upon loss of ΔΨ, PINK1 import and cleavage are blocked, allowing the kinase to accumulate on the outer membrane [278], where it phosphorylates ubiquitin [279] with the E3 ligase Parkin [280], thereby activating Parkin-dependent ubiquitylation of outer membrane substrates. A long mitochondrial isoform of PTEN (PTEN-L) dephosphorylates Ser65-phosphorylated ubiquitin and acts as a negative regulator of this pathway [281]; ROS-mediated PTEN inactivation [282] may therefore relieve this brake and facilitate PINK1/Parkin-dependent MDV formation under oxidative stress [274].

A second organelle-level QC mechanism segregates damaged material through asymmetric fission. Live-cell imaging in β-cells and other systems shows that many fission events generate unequal daughters: one exhibits high membrane potential and a high probability of re-fusion with the network, whereas the other displays reduced ΔΨ, loss of fusion factors such as OPA1, and an increased likelihood of mitophagic capture [283]. This “peripheral fission” is often initiated at sites of contact with lysosomes [284], in contrast to midzone fission events that are pre-constricted by ER and actin and primarily serve biogenesis. When mitochondrial damage is extensive and depolarisation sustained, the PINK1–Parkin cascade marks these low-ΔΨ fragments for elimination by ubiquitylating outer membrane proteins including mitofusin 1 [285] and 2 [285], Miro1 [286], and VDAC1 [287]. Ubiquitin chains on the outer membrane then recruit autophagy adaptors and the core autophagic machinery, culminating in mitophagy [288]. In parallel with this canonical PINK1/Parkin route, several receptor-mediated and Parkin-independent mitophagy pathways operate under specific developmental or stress conditions, as reviewed elsewhere [289].

Beyond MDVs and mitophagy, recent work in differentiating neural progenitors has uncovered a more drastic mitochondrial “suicide” programme in which transient fusion between the mitochondrial outer membrane and the nuclear envelope allows nuclear-encoded RNAs to enter the intermembrane space [290]. These RNAs are degraded by mitochondrial polynucleotide phosphorylase, releasing sequestered protons [290,291] and acutely increasing ΔpH and ROS production, which drives oxidative protein damage and progressive mitochondrial dysfunction. Subsequent dissipation of ΔpH, thermogenesis, and the liberation of single-stranded mtDNA segments further undermine mitochondrial transcription via DNA–RNA hybrid formation and RNase H1-mediated degradation of transcripts, thereby complementing ROS-mediated protein damage and locking the organelle into an irreversible degenerative trajectory [248]. In contrast to the graded, repair-oriented functions of MDVs and peripheral fission, this pathway represents a terminal QC option in which mitochondria actively participate in self-elimination when damage exceeds salvageable limits.

Together, MDVs, asymmetric fission–mitophagy, and such extreme “suicide” programmes illustrate how, at higher tiers of the cascade dynamics, mitochondrial QC shifts from local repair of damaged components to selective, and ultimately wholesale, removal of organelles that have crossed a functional point of no return. When mitochondrial QC processes (proteostasis, fusion–fission balance, and mitophagy) can no longer efficiently remove or repair dysfunctional organelles, there is progressive build-up of mitochondria with depolarized membranes, high ROS output, and elevated matrix Ca^2+^. These changes promote opening of the mitochondrial permeability transition pore (PTP), a Ca^2+^- and ROS-sensitive channel whose activation causes loss of membrane potential, osmotic swelling of the matrix, and rupture of the outer mitochondrial membrane [292,293,294]. Outer membrane rupture allows release of intermembrane space proteins such as cytochrome c, Smac/DIABLO, and apoptosis-inducing factor (AIF) into the cytosol, where cytochrome c associates with Apaf-1 and dATP to form the apoptosome, leading to sequential activation of caspase-9 and effector caspases such as caspase-3 [292,293,294]. Anti-apoptotic Bcl-2 family proteins inhibit this progression by stabilising mitochondrial membranes and suppressing PTP opening, but when mitochondrial damage exceeds QC capacity and pro-apoptotic signals dominate, a growing fraction of the mitochondrial population undergoes permeability transition, amplifying caspase activation and driving the cell irreversibly into the execution phase of apoptosis [294]. It is noteworthy that while in classical “catastrophic” mode sustained PTP opening causes full dissipation of the inner membrane potential, matrix swelling, outer membrane rupture, and release of apoptogenic factors, flickering events have also been reported whereby low-conductance, short-duration openings allow limited equilibration of small ions and solutes across the inner membrane but are rapidly followed by pore closure and re-establishment of the membrane potential [295,296]. Functionally, flickering mPTP activity is proposed to act as a “safety valve” by releasing small amounts of Ca^2+^ from the matrix back to the cytosol, helping prevent sustained mitochondrial Ca^2+^ overload. Transient PTP also modulates ROS levels by allowing partial dissipation of the proton motive force and transient changes in redox state, thereby preventing runaway ROS production.

## 4. Signalling Network Topology of Mitochondrial QC Mechanisms

**Figure 4:**
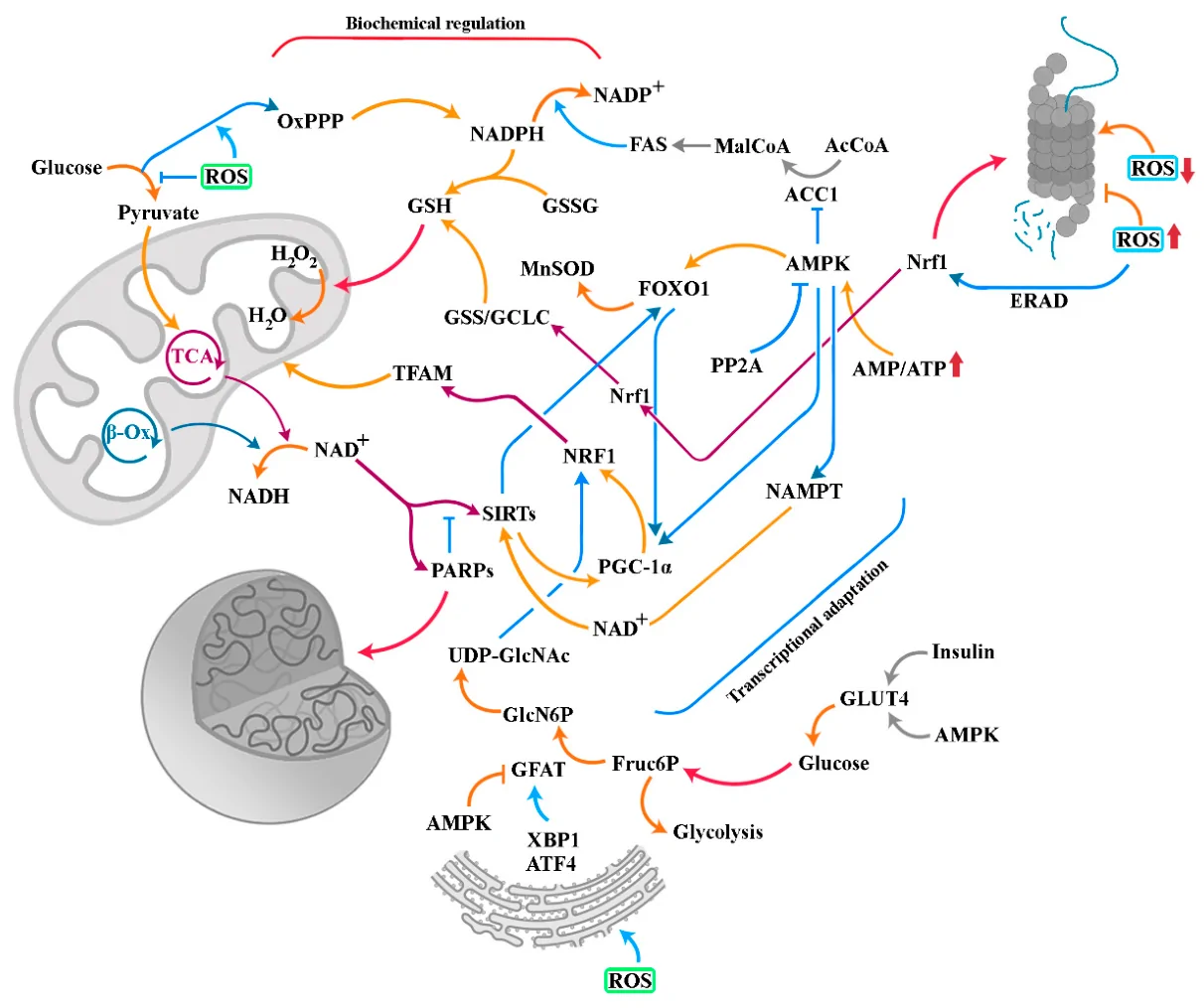
Integrated biochemical and transcriptional control of mitochondrial stress responses and quality control. Glucose-derived metabolites feed into the oxidative branch of the pentose phosphate pathway (oxPPP) to generate NADPH, which maintains the glutathione pool in its reduced form (GSH) via GSSG/GCLC and supports detoxification of mitochondrial ROS emerging from the ETC and β-oxidation. Energy and redox status, reflected in AMP/ATP and NAD^+^/NADH ratios, activate AMPK and sirtuins, which converge on NAMPT, PGC-1α and NRF1 to promote NAD^+^ salvage, mitochondrial biogenesis and transcriptional adaptation. Parallel regulation of glucose uptake (GLUT4), glycolysis, and the hexosamine pathway (GFAT, UDP-GlcNAc) through AMPK, XBP1 and ATF4 links cytosolic metabolic reprogramming to organellar QC. Endoplasmic reticulum-associated degradation (ERAD) and Nrf1 further integrate proteostatic stress with mitochondrial ROS signalling, such that biochemical regulation (NADPH, glutathione and lipid synthesis) and transcriptional adaptation (AMPK–Sirt–PGC-1α–NRF1/TFAM axes) together shape the cascade-like mitochondrial quality control response to oxidative and metabolic stress.

The signalling network that coordinates mitochondrial QC integrates inputs from three metabolic layers, beginning with the partitioning of glucose between glycolysis and the hexosamine biosynthetic pathway (HBP) (Figure 4). Membrane localization of Glut4 triggered by insulin-mediated activation of the PI3K/AKT pathway [297] and reinforced by AMPK signalling [298,299] results in intracellular accumulation of glucose. At the fructose-6-phosphate branch point, this glucose can either enter glycolysis or be channelled into the HBP, which uses glucose and glutamine to generate UDP-GlcNAc, the donor substrate for O- and N-linked protein glycosylation. HBP flux is largely governed by the rate-limiting enzyme GFAT1 (glutamine:fructose-6-phosphate amidotransferase-1): AMPK-mediated phosphorylation inhibits GFAT1 activity [300], whereas ER-stress pathways such as XBP1s [301] and GCN2–ATF4 [302] upregulate GFAT1 expression during the unfolded-protein response. Because oxidative stress perturbs ER redox homeostasis and protein folding [303], an oxidising milieu that challenges ER function tends to increase HBP flux and UDP-GlcNAc synthesis, thereby enhancing glycosylation capacity.

Elevated UDP-GlcNAc enables stress-responsive glycosylation events that directly influence mitochondrial QC. O-GlcNAcylation of HSF1 and Sirt1 potentiates activity and reduces mitochondrial Ca^2+^ uptake and ROS generation, while a cycle of N-linked glycosylation and subsequent de-glycosylation edits specific N-glycosylated Asn residues within Nrf1 (NFE2L1) to Asp, a modification required for full transcriptional activation [158]. Activated Nrf1 then translocates to the nucleus and binds antioxidant response elements in target promoters, including those of GCLM [304,305] (the modifier subunit of glutamate–cysteine ligase) and GSS [306,307] (glutathione synthetase), thereby increasing glutathione synthesis. The GCL holoenzyme, composed of catalytic (GCLC) and modifier (GCLM) subunits, catalyses the rate-limiting step in GSH biosynthesis; GCLM lowers the Km for glutamate and raises the Ki for feedback inhibition by GSH [308], ensuring efficient GSH production under stress. Thus, ER-stress-driven HBP activation, ER-associated degradation (ERAD) sensing, and glycosylation-dependent Nrf1 maturation collectively couple glucose availability and ER proteostasis to the size and responsiveness of the cellular GSH pool, thereby priming the mitochondrial QC network for operation in an oxidising environment.

In this framework, glucose influx is interpreted as a potential stress signal only when considered alongside ER stress inputs such as oxidative disruption of ER folding capacity [309] or PERK–eIF2α activation by mitochondria [310] which engage Nrf1-dependent antioxidant and proteasome programmes. Because the ER relies on an oxidising redox potential for disulfide-bond formation, whereas mitochondrial QC benefits from a more reducing environment, Nrf1-mediated upregulation of proteasome components and glutathione synthesis can be viewed as an adaptive strategy to reconcile this redox trade-off and maintain both ER proteostasis and mitochondrial resilience under fluctuating nutrient and redox conditions [311].

While ER-resident Nrf1 interprets oxidative stress through the lens of ER proteostasis, cytosolic Nrf2 responds more directly to changes in the cellular redox environment [312]. Under oxidative stress, Nrf2 is released from its inhibitor Keap1, accumulates in the nucleus, and binds antioxidant-response elements (AREs) in promoters of target genes including GCLM, GCLC, glutathione peroxidase-2 (GPX2), various glutathione S-transferases, and heme oxygenase-1 [313,314]. In differentiated cells, Nrf2-dependent programmes complement those of Nrf1 by further expanding glutathione synthesis and phase-II detoxification capacity [315], whereas in cycling cells the relative contributions of Nrf1 and Nrf2 to antioxidant defence and proteostasis diverge during development [316]. In parallel, insulin signalling augments GSH synthesis by activating GCLC via IRS–PI3K [317] and Raf–MEK–ERK [318] pathways, effectively pre-conditioning cells for ROS production that may follow glucose uptake.

The expanded GSH pool must be matched by sufficient NADPH to reduce GSSG back to GSH. NADPH-dependent GSH regeneration is largely supplied by the oxidative branch of the pentose phosphate pathway (oxPPP) [319]. Upon a shift to an oxidising environment, key lower-glycolytic enzymes such as glyceraldehyde-3-phosphate dehydrogenase [320] and pyruvate kinase [74] are selectively inactivated, diverting flux from upper glycolysis into the oxPPP and increasing NADPH output. ROS can also activate the oxPPP via oxidation of the kinase ATM, promoting intermolecular disulfide bond formation and dimerization [321] independently of DNA damage [322]. Activated ATM phosphorylates Hsp27, which associates with and stimulates the rate-limiting oxPPP enzyme glucose-6-phosphate dehydrogenase (G6PD), further boosting NADPH production [323]. This rapid, post-translational ROS-driven activation of G6PD is complemented by slower transcriptional upregulation of oxPPP components, providing both an immediate and sustained increase in reducing power [324].

The regulatory logic underlying GSH upregulation therefore exemplifies a general pattern of mitochondrial adaptation: a fast layer of post-translational control followed by a delayed transcriptional response. MnSOD, which converts superoxide radicals to H_2_O_2_, follows a similar biphasic scheme. Enhanced MnSOD activity can be achieved acutely by denitration of the enzyme, a process that is promoted under hypoxia and thought to precondition mitochondria for subsequent reoxygenation-induced ROS bursts [212]. On a longer timescale, stress-activated NF-κB serves as a trans-activator of MnSOD [213,214] and simultaneously induces thioredoxin-1 and thioredoxin-2 [325], thereby strengthening both superoxide dismutation and thiol-disulfide redox buffering in response to oxidative challenges [215,216,326].

Beyond redox chemistry, secondary consequences of oxidative stress feed into energy-sensing pathways that further remodel mitochondrial QC. iPLA_2_γ-mediated release of free fatty acids from oxidised phospholipids promotes fatty acid-dependent proton leak via mitochondrial uncoupling proteins [236] and adenine nucleotide translocase [238,239,240], leading to dissipation of the proton motive force (Δp) and matrix acidification. In response, ATP synthase can operate in reverse, hydrolysing ATP to pump protons out of the matrix and restore pH [31], thereby increasing ADP and raising the AMP:ATP ratio, which scales approximately with the square of the ADP:ATP ratio [34]. In parallel, activation of the oxPPP contributes to AMPK signalling by relieving protein phosphatase 2A-mediated inhibition of AMPK [327], further sensitising the kinase to energetic stress.

Once activated, AMPK enhances mitochondrial antioxidant capacity through several convergent mechanisms. AMPK-mediated phosphorylation of FoxO1 promotes FoxO1-dependent transcription of MnSOD [48], while AMPK-driven activation of PGC-1α increases expression of MnSOD and other mitochondrial antioxidant genes [49]. AMPK also boosts Sirt1 activity by increasing cellular NAD^+^ levels [81], and Sirt1 in turn deacetylates PGC-1α [82] to further augment its transcriptional coactivator function and MnSOD expression. In the matrix, Sirt3 deacetylates and activates IDH2, increasing mitochondrial NADPH and the GSH:GSSG ratio, and also deacetylates MnSOD to raise the catalytic efficiency. Intriguingly, AMPK-dependent phosphorylation enhances PGC-1α-driven activation of its own promoter, establishing a positive feedback loop [328]. Together, these interactions create an AMPK–Sirt1/3–PGC-1α feed-forward module that is initiated by ROS-driven changes in Δp and matrix pH and culminates in coordinated upregulation of MnSOD, NADPH production, and GSH regeneration. In this way, ROS-driven activation of AMPK [329] translates secondary stressors such as mitochondrial acidification into a reinforced antioxidant and QC programme that counteracts the original oxidative insult.

Activation of AMPK during adaptation to oxidative stress also restrains de novo lipogenesis, a process that consumes large amounts of NADPH [330], the same reducing currency required for glutathione regeneration. Each cycle of fatty acid elongation uses two molecules of NADPH; thus, synthesis of a typical C16 fatty acid from acetyl-CoA requires 14 NADPH equivalents per molecule [330]. By phosphorylating and inhibiting acetyl-CoA carboxylase-1 (ACC1) at a conserved serine residue, AMPK acutely suppresses lipogenic flux and limits fatty acid synthesis [331,332]. In the context of oxidative stress, this AMPK-mediated inhibition of lipogenesis is expected to spare NADPH for competing antioxidant reactions, particularly the reduction in oxidised glutathione, thereby reinforcing mitochondrial redox defences. This reduced consumption of NADPH in lipogenesis complements ROS-mediated inactivation of key lower-glycolytic enzymes such as glyceraldehyde-3-phosphate dehydrogenase [320] and pyruvate kinase [74], which diverts flux from upper glycolysis into the oxPPP, increasing the ratio of NADPH:NADP^+^. The resulting positive feedback loop, in which activation of the oxidative PPP promotes AMPK signalling by relieving protein phosphatase 2A-mediated inhibition of AMPK [327], further increases the response capacity of this adaption. Finally, AMPK supports mitochondrial antioxidant capacity by phosphorylating FoxO1 to drive MnSOD expression [48] and, via PGC-1α, by increasing MnSOD and Sirt3 activity [49].

Notably, AMPK activation does not strictly require a global energetic crisis. In the context of nutrient excess, increased glucose uptake and ROS production can activate AMPK [329] through changes in mitochondrial ATP output, local ADP/AMP microdomains, and upstream kinases such as LKB1 and CaMKKβ [333], even when bulk ATP levels remain within the physiological range. In this way, glucose- and ROS-driven signals engage AMPK as a metabolic integrator long before catastrophic energy failure occurs, allowing the kinase to pre-emptively remodel redox buffering, lipogenesis, and mitochondrial QC programmes.

Transcriptional adaptation of mitochondria to stressors is supported by nuclear respiratory factor-1 (NRF1), which acts in parallel and partially synergistically with the ER-resident transcription factor Nrf1/NFE2L1 [334]. NRF1 (also known as α-Pal) partners with PGC-1α to activate expression of mitochondrial transcription factors such as TFAM, TFB1M, and TFB2M, as well as a wider set of nuclear-encoded OXPHOS subunits and import components [335,336]. Two metabolic sensors, AMPK and Sirt1, directly modulate PGC-1α activity: AMPK phosphorylates PGC-1α [328], and Sirt1 deacetylates specific lysine residues in an NAD^+^-dependent manner [82], together shifting PGC-1α into a more transcriptionally active state [337]. Downstream of AMPK–Sirt1/3 signalling, PGC-1α coactivates NRF1 to induce a broad programme that includes respiratory-chain subunits, heme biosynthetic enzymes, mitochondrial transcription and import factors such as TFAM and TOM/TIM components, and oxidative metabolism genes. TFAM, in particular, is central to mitochondrial adaptation to stress because of key roles in mtDNA transcription, replication, and nucleoid compaction [338]. This NRF1/PGC-1α module therefore couples redox and energetic cues to mitochondrial biogenesis and replenishment of mitochondrial QC machinery.

Another signalling cascade that contributes to mitochondrial QC and is regulated by combined AMPK and Sirt1 activity is the FOXO (Forkhead box O) pathway. Growth factor-driven PI3K–AKT signalling phosphorylates FOXO proteins [339] and promotes their export from the nucleus [340], whereas stress-activated JNK [341], AMPK [342], and Sirt1 [343,344] favour FOXO nuclear retention and trans-activation of genes encoding MnSOD, catalase, thioredoxins, autophagy factors, and metabolic enzymes that enhance mitochondrial antioxidant capacity and quality control [345,346]. In parallel, FOXO transcription factors enforce cell-cycle checkpoints by inducing cyclin-dependent kinase inhibitors such as p27^Kip1^ and p21^Cip1^ [347], repressing G1/S cyclins [348], and regulating replication factors including Cdt1 and CTDSP2 [349], thereby linking oxidative stress and AMPK/Sirt1 signalling to proliferation control. In this manner, mitochondrial QC is tied to cell-cycle progression, a connection that is explored in a subsequent section.

The emerging picture suggests that AMPK and Sirt1 orchestrate both short-term (phosphorylation and deacetylation) and longer-term (transcriptional) adaptations that amplify mitochondrial QC. AMPK also functions as an upstream collaborator of Sirt1. Sirt1 consumes NAD^+^ and generates nicotinamide (NAM), which feeds back as a product inhibitor [350], so continuous Sirt1 activity requires efficient reconversion of NAM into NAD^+^ via the salvage pathway. The rate-limiting enzyme in this pathway is nicotinamide phosphor-ribosyl-transferase (NAMPT) [351]. AMPK upregulates NAMPT expression [49] and directly phosphorylates NAMPT to enhance catalytic activity [352], thereby increasing NAD^+^ availability and promoting Sirt1 activation. The resulting activation of FOXO and PGC-1α signalling then engages the suite of mitochondrial QC mechanisms outlined above. PGC-1α-dependent transcription not only enhances mitochondrial QC capacity but also promotes mitochondrial biogenesis, in part through NRF1-TFAM-driven replication of mtDNA and expansion of the organelle pool. PGC-1α-driven replication can also enter a “quasi-replication” mode under conditions of high membrane potential and thermogenic output, in which replication of the parental light strand predominates, liberating single-stranded H-strand segments and contributing to transcriptional shut-down of mitochondria [248]. In this model, the branch point between full replication and quasi-replication is governed by mitochondrial Δp and heat production, with hyperpolarised, highly thermogenic mitochondria favouring quasi-replication.

While a high mitochondrial membrane potential (Δp) amplifies ROS production, dissipation of the proton gradient in response to oxidative stress increases proton leakage and thermogenesis, thereby introducing heat as a secondary stressor [353]. A major strategy deployed by mitochondria to adapt to heat is the recruitment of heat-shock proteins (HSPs), as discussed earlier [354]. However, the effects of heat-induced activation of HSP27 [355] extend well beyond organellar acclimation. In this context, the mutually reinforcing relationship between heat shock factor 1 (HSF1) and oxidative stress is particularly noteworthy. Both heat and oxidising agents activate HSF1 via homo-trimerisation [141], and activated HSF1 binds promoters and enhancers of genes that regulate cellular redox homeostasis, thereby trans-activating their transcription [356]. In a more specific interaction, Hsp27 associates with and increases the activity of glucose-6-phosphate dehydrogenase (G6PDH), the rate-limiting enzyme of the oxidative branch of the pentose phosphate pathway, enhancing NADPH production and antioxidant capacity [323]. HSF1 also elevates cellular NAD^+^ levels by modulating the enzymes of the NAD^+^ salvage pathway [357]. The resulting Sirt1-mediated deacetylation amplifies HSF1 signalling activity [79] and, as discussed earlier, boosts mitochondrial antioxidant defences.

HSF1 further impacts mitochondrial genome maintenance. HSF1 suppresses the oligomerisation of single-stranded DNA-binding protein 1 (SSBP1) [255], which is critical for high-affinity binding to DNA and for the regulation of mtDNA replication. Together with the inherent thermolability of mitochondrial SSBP1 (mtSSBP1) [248], this suppression reduces its DNA-binding capacity and exposes the heavy strand (H-strand) of mitochondrial DNA. These exposed H-strand regions then act as natural antisense templates that base-pair with complementary heavy-strand mRNAs to form DNA–RNA hybrids, which are subsequently processed by RNase H1 [248]. Degradation of these hybrids reduces mitochondrial transcript levels, dampens the electron transport chain assembly and, consequently, limits further ROS and heat production.

In mitochondria, conversion of primary stressors into secondary stress signals is not merely dissipative; it serves to integrate multiple QC modules into a coherent adaptive response. Elevated membrane potential and electron leakage, for example, generate reactive oxygen species as a primary signal, while partial uncoupling dissipates Δp and converts part of this oxidative burden into heat, which in turn activates heat-shock and HSF1-dependent transcriptional programmes. Oxidative stress also modifies lipids, proteins and mitochondrial DNA, recruiting mitophagy, proteostasis networks and nucleoid-level QC pathways that operate on longer timescales. Through these cascades, a single initiating perturbation is progressively transformed into a spectrum of qualitatively distinct stress signals, each of which engages partially non-overlapping surveillance and repair mechanisms. The cumulative effect is to dilute the impact of any one primary insult while expanding the repertoire of adaptive options available to the organelle.

Importantly, the pathways that respond to secondary stressors often feedback positively on defences against the original insult. Heat-induced activation of HSF1 and HSPs enhances antioxidant capacity and stabilises components of the respiratory chain, thereby limiting further ROS generation. Likewise, AMPK–Sirt1 signalling triggered by energetic stress increases NAD^+^ availability, augments mitochondrial antioxidant systems and promotes biogenesis of more fit organelles, which restores redox balance and membrane potential. In this way, the cascade-like conversion of stressors not only attenuates primary damage but also creates a network of reinforcing feedback loops that stabilise mitochondrial function across a range of physiological and pathological conditions.

## 5. Mitochondrial QC in the Context of Cell Cycle and Differentiation

**Figure 5:**
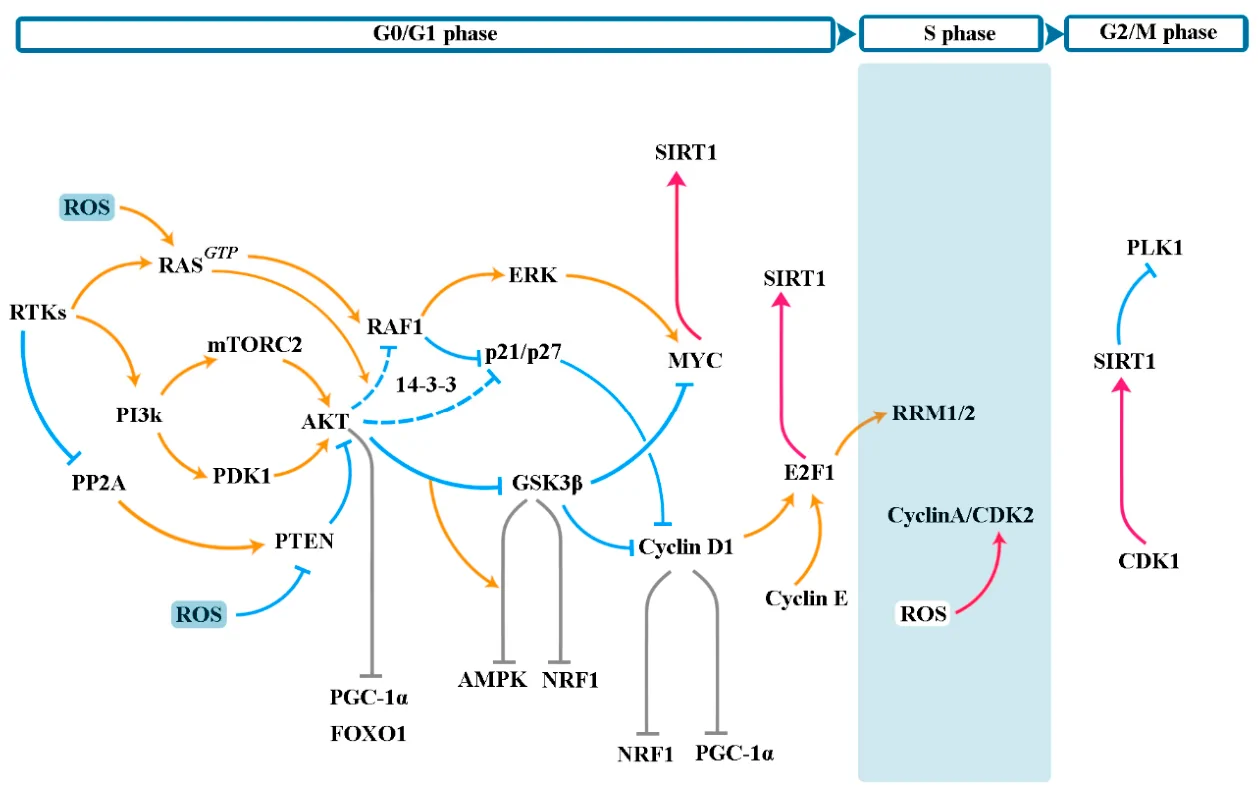
Cell-cycle-dependent integration of mitogenic signalling, Sirt1 and mitochondrial quality control regulators. Schematic representation of key pathways that coordinate progression from G0/G1 through S phase to G2/M with transcriptional and metabolic control of mitochondrial QC. In early G1, receptor tyrosine kinase signalling activates the RAS–PI3K–PDK1–AKT and mTORC2 cascades, which converge on RAF1, p21/p27 and 14-3-3 to regulate ERK and GSK3β. AKT, GSK3β and cyclin D1 collectively exert inhibitory effects on mitochondrial QC components such as AMPK, NRF1 and PGC-1α (grey arrows), thereby prioritising anabolic growth programmes over stress adaptation. As cells progress toward late G1, increasing activity of E2F1 and c-Myc reverses this balance, activating Sirt1, NRF1 and PGC-1α (red arrows) and promoting transcriptional programmes that enhance mitochondrial biogenesis and antioxidant capacity. Entry into S phase is accompanied by E2F1-dependent induction of ribonucleotide reductase (RRM1/2) and cyclin A/CDK2, alongside rising ROS levels, while in G2/M, Sirt1 modulates CDK1 and PLK1 to fine-tune mitotic progression and mitochondrial output. Together, these interactions define a late-G1 window in which mitogenic cues, ROS signalling and Sirt1-dependent transcription jointly configure mitochondrial QC for proliferating cells.

The generic components of the mitochondrial QC network described herein operate within the biological constraints imposed by the cell cycle (Figure 5). In cycling cells, these constraints largely reflect the temporal segregation of key signalling pathways into distinct cell-cycle phases. A central regulator of progression through G1 is the c-Myc proto-oncogene, a pro-anabolic transcription factor that promotes growth and proliferation [358]. To support the anabolic demands of cycling cells, c-Myc stimulates ribosome biogenesis and enhances global protein synthesis during G1 [359]. In parallel, c-Myc binds the Sirt1 promoter and activates Sirt1 expression [360]. Sirt1, in turn, deacetylates c-Myc, reducing its stability and creating a negative feedback loop that aligns Sirt1-dependent mitochondrial activity with c-Myc-driven anabolic flux in G1 [360].

Progression from G1 into S phase requires additional input from the E2F pathway. A feedback motif analogous to that of c-Myc governs the interaction between Sirt1 and E2F1: E2F1 activates Sirt1 transcription, whereas Sirt1 subsequently inhibits E2F1 activity [361]. Beyond transcriptional control, E2F1 may further enhance Sirt1 by modulating its post-translational stability. Inactivation of the anaphase-promoting complex/cyclosome APC/C-Cdh1 represents the critical commitment step after which stimulated cells proceed towards S phase [362]. E2F1 contributes to APC/C-Cdh1 inactivation by inducing expression of the inhibitor, Emi1 [363]. E2F1-mediated suppression of APC/C-Cdh1 diminishes the polyubiquitination of Sirt1 by this complex and thus limits proteasomal degradation [364]. Consequently, Sirt1-dependent control of mitochondrial QC mechanisms is expected to become more prominent towards late G1. Consistent with this notion, cellular ROS levels have been reported to increase progressively from G1 into S phase and further into G2 [365,366].

Additional layers of regulation converge on this temporal window. Cyclin D1, an upstream activator of E2F transcription factors, inhibits the transcription factor Nrf1 [367], thereby constraining mitochondrial biogenesis early in G1. This inhibitory influence is relieved when cyclin D1 is polyubiquitinated by the Skp1–Cul1–F-box (SCF) ubiquitin ligase complex and targeted for degradation [368], a process that coincides with rising SCF activity in late G1 [369]. Taken together, these observations support the hypothesis that the interval between E2F1 activation and cyclin D1 degradation constitutes a window during which mitochondrial QC pathways are preferentially upregulated. This idea is in line with the finding that aerobic glycolysis in cycling cells is induced within a specific temporal window between APC/C-Cdh1 inactivation and SCF-β-TrCP activation [370], suggesting coordinated timing of metabolic reprogramming and mitochondrial QC.

The proposal that mitochondrial QC is regulated in a cycle-dependent manner during late G1 is based on isolated lines of evidence derived from different model systems and experimental approaches that are only partly aligned with cell-cycle dynamics. For example, although E2F1-mediated upregulation of Sirt1 is supported by rigorous observations, that study focused on apoptosis rather than the cell cycle [361]. Complementing the activity of E2F1, c-Myc activates Sirt1 by two parallel mechanisms [371]. Upregulation of NAMPT by c-Myc, the rate-limiting enzyme of the NAD^+^ salvage pathway, increases the amount of NAD^+^. In parallel, c-Myc contributes to Sirt1 activation by sequestering the Sirt1 inhibitor deleted in breast cancer 1 (DBC1) [371]. A key challenge in validating the idea that mitochondrial QC is regulated in late G1 is the development of model systems that can track progression through G1 at multiple levels and define “late G1” with greater precision. This would typically require integrating transcriptional profiling of a cycling population with translational and post-translational analyses. Maintaining synchrony within the cycling population is another important confounding factor that must be considered.

Beyond cycling cells, acquisition of a differentiated phenotype could reshape the regulation of the mitochondrial QC network. The functional specialisation of brown adipose tissue illustrates this principle. Thermogenesis in brown adipocytes is driven by uncoupling protein 1 (UCP1), which is activated by long-chain fatty acids released from lipolysis of cytoplasmic lipid droplets upon adrenergic stimulation [372]. Although UCP1 is expressed in several cell types [373], its activity is particularly high in brown adipose tissue because of privileged access to the cytosolic pool of long-chain fatty acids [372]. As a result, UCP1-mediated uncoupling maintains a relatively low mitochondrial membrane potential, limiting electron leak and thereby reducing ROS production [374]. In this setting, the primary mitochondrial output of the cascade is heat rather than ROS, and QC adaptations are tuned accordingly.

In contrast, mitochondrial ROS production in skeletal muscle is enhanced by the high membrane potential that develops under many physiological conditions, leading to over-reduction of the electron transport chain and increased superoxide formation [375]. This amplified ROS generation is counterbalanced by robust and dynamically regulated antioxidant defences, including superoxide dismutase, catalase and glutathione peroxidase, which are further upregulated by contractile activity and exercise [376]. Here, mitochondrial QC relies more heavily on inducible antioxidant capacity to accommodate large, activity-dependent swings in ROS flux.

A second example of differentiation-dependent rewiring of mitochondrial QC comes from hepatic NADPH metabolism. Whereas most tissues rely predominantly on the oxidative branch of the pentose phosphate pathway (oxPPP) to produce NADPH, the liver can generate substantial amounts of NADPH via folate-dependent serine catabolism [377]. This reduced dependence on oxPPP for NADPH supply alters how ROS are integrated into the mitochondrial QC network, shifting some of the burden from PPP-linked redox control to one anchored in one-carbon metabolism. Collectively, these examples illustrate that the cascade dynamics of mitochondrial QC acquire an additional layer of sophistication in differentiated tissues, as primary stressors are converted into distinct secondary signals and buffered by tissue-specific metabolic architectures.

## 6. Mitochondrial QC Across Organ Systems

At an organ level, the mitochondrial QC network is regulated by organ-level multicellular interactions and endocrine hormones. Although a comprehensive treatment of this topic lies beyond the scope of this review, we provide a brief overview of some regulatory influences here.

To delineate the impact of organ-level cellular interactions on mitochondrial QC, we focus on intercellular communication in the central nervous system. As discussed earlier, the pentose phosphate pathway (PPP) protects neurons against oxidative stress [378]. Notably, astrocytes divert glucose into the PPP at rates five- to sevenfold higher than neurons [379]. Under hypoxic conditions, which are known to induce ROS [380], glucose flux through the PPP increases in astrocytes but decreases in neurons, underscoring the importance of functional complementation within multicellular organs. Astrocytes also export GSH to the extracellular space, thereby supplying neighbouring neurons with this potent antioxidant [381]. Paracrine support of neuronal function is not limited to these examples: astrocytes secrete exosomes enriched in nicotinamide phosphoribosyltransferase (Nampt), which are taken up by adjacent neurons and increase NAD^+^ levels, leading to AMPK activation and mTOR inhibition [382]. Together, these examples of functional complementation illustrate how organ-level cellular interactions can modulate cell-intrinsic mitochondrial QC mechanisms in a paracrine manner. Beyond paracrine signalling, the endocrine system provides an additional layer of regulation of mitochondrial QC, with thyroid hormones serving as a prime example of this interaction.

Thyroid hormones modulate the mitochondrial QC network via two interconnected mechanisms [383]: (i) receptor-independent actions on the mitochondrial electron transport chain (ETC) and inner membrane, and (ii) receptor-dependent transcriptional programmes driven predominantly by T3 (triiodothyronine) binding to nuclear thyroid hormone receptors TRα and TRβ [384]. Through nongenomic effects, T3 can acutely enhance oxidative phosphorylation within minutes, increasing electron flux and proton leak, which secondarily amplifies mitochondrial reactive oxygen species (ROS) production [385,386,387]. Consistent with this, hyperthyroid liver exhibits mitochondrial ultrastructural abnormalities, including outer membrane rupture and reduced cristae density, alongside increased GSSG/GSH ratios and extensive mitochondrial DNA damage [388,389]. To counteract these injurious consequences, thyroid hormones engage mitochondrial QC pathways that integrate biogenesis, dynamics, and mitophagy. In skeletal muscle, T3 upregulates expression of the upstream AMPK kinase LKB1 and its scaffold partner MO25, leading to increased AMPK phosphorylation and activity in the hyperthyroid state [390,391]. AMPK activation elevates NAD^+^ availability and stimulates Sirt1, which in turn activates PGC-1α [392], a central transcriptional coactivator governing mitochondrial biogenesis and functional adaptation [393]. Sirt1 also acts as a direct, gene-selective coactivator of TRβ1, physically interacting with the receptor, promoting its deacetylation and turnover, and enhancing T3-responsive transcription at specific metabolic target genes in the liver [394]. This interaction suggests a feed-forward regulatory loop where Sirt1 potentiates receptor-dependent thyroid hormone signalling in the liver. Such receptor-mediated signalling generally support mitochondrial QC and stress adaptation, partially offsetting the direct, receptor-independent pro-oxidant effects of thyroid hormones [383]. Similarly to the impact of thyroid hormones on skeletal muscle, leptin stimulates phosphorylation and activation of AMPK in skeletal muscle, thereby suppressing the activity of ACC (acetyl-CoA carboxylase) stimulating the oxidation of fatty acids [395]. Therefore, endocrine signals constitute an additional hierarchical layer regulating mitochondrial quality control.

## 7. Conclusions

In this review, we describe the organisation of mitochondrial stress responses and quality control (QC) at the organellar level, from which several principles emerge.

We suggest that mitochondria actively interpret stress through their bioenergetic and biosynthetic functions, converting primary perturbations into secondary signals such as ROS, heat, mtDNA damage, and metabolic rewiring. These cascades broaden the range of QC pathways engaged. In parallel, stress-induced reprogramming—via axes such as AMPK–Sirt1–NAMPT, HSF1-dependent proteostasis, and PGC-1α-driven biogenesis—coordinates adaptive changes in respiration, antioxidant capacity, mitophagy, and genome maintenance, highlighting QC as a dynamic network. Importantly, these responses are selective. We propose a “cascade effect,” whereby identical stressors generate distinct outputs depending on mitochondrial state, cell-cycle phase, and differentiation context.

Together, this framework positions mitochondria as integrators of stress, with QC operating through cascade-like signal conversion. This perspective has implications for disease and suggests that effective interventions may need to target both QC pathways and the processes that shape stressor conversion.

## 8. Open Questions and Future Direction

This synthesis advances a view of mitochondrial QC as a cascade-like, selectively engaged network that converts primary stressors into secondary signals and integrates them across cell-cycle stages and differentiated tissues. Several key questions arise from this framework.

First, the molecular logic of stressor conversion remains incompletely defined. How do specific features of the mitochondrial state—such as membrane potential, substrate availability, and respiratory complex composition—determine whether a given insult is channelled predominantly into ROS, heat, mtDNA damage or metabolic reprogramming? Is it possible to identify discrete “conversion nodes” (for example, UCP1, ANT, or particular dehydrogenases) whose modulation is sufficient to re-route stressor cascades and thereby reprogramme QC outputs? Addressing these questions will require quantitative, time-resolved measurements of primary and secondary stress signals in intact cells and tissues, ideally combined with computational models that can predict cascade behaviour under physiological and pathological conditions.

Second, many of the proposed feedback loops linking stress-sensing to QC—such as the AMPK–Sirt1–NAMPT axis, HSF1/HSP-dependent reshaping of redox metabolism, and PGC-1α-driven control of mitochondrial replication and quasi-replication remain only partially mapped at the mechanistic level. It will be important to test, in a causal manner, whether selective disruption of these loops alters the hierarchy of QC responses, or simply changes their magnitude. For example, does perturbing Sirt1-dependent regulation of PGC-1α or HSF1 shift mitochondria toward repair, replacement, or shutdown modes of QC in response to identical primary stressors? Similarly, does experimentally enforcing or preventing quasi-replication of mtDNA measurably change susceptibility to chronic or intermittent stress?

Third, the proposal developed herein that mitochondrial QC is temporally gated by the cell cycle raises several experimentally tractable questions. Does the “late-G1 QC window” suggested by c-Myc/E2F/Sirt1/Nrf1 dynamics exist across diverse cell types, or is it restricted to particular proliferative contexts? How do cell-cycle checkpoints and DNA damage responses intersect with mitochondrial stress cascades, for example, via RNR-dependent dNTP supply, ROS-sensitive checkpoint kinases, or mitotic control of mitochondrial distribution and turnover? Single-cell approaches that track cell-cycle phase, mitochondrial state and QC readouts in parallel will be essential to test these ideas.

Fourth, differentiation imposes tissue-specific architectures on mitochondrial QC, as illustrated by UCP1-driven uncoupling in brown adipocytes, high-Δp-dependent ROS signalling and inducible antioxidant systems in skeletal muscle, and folate-mediated NADPH production in liver. A major challenge is to systematically define how these specialised configurations reshape stressor cascades and their downstream QC modules. Do different tissues occupy distinct “QC regimes,” with characteristic balances between repair, mitophagy, biogenesis and functional shutdown, and can these regimes be re-programmed in disease or ageing? Comparative analyses across tissues and between developmental stages should clarify how universal the proposed principles are, and where organ-specific exceptions occur.

Finally, this conceptual framework has implications for therapeutic intervention. If mitochondria act as selective integrators of stress, then targeting individual QC components may be less effective than modulating the conversion steps that shape the stressor cascade itself. Future work should explore whether controlled manipulation of Δp, ROS signalling nodes, NAD^+^/NADPH metabolism, or mtDNA replication modes can “re-tune” mitochondrial QC to favour adaptive outcomes in metabolic disease, neurodegeneration, cancer and ageing. Realising this potential will require integrating organellar-level mechanistic studies with in vivo models and human data, to determine when and where altering cascade dynamics is both feasible and beneficial.

## Figures and Tables

**Table 1:** Table shows redox-mediated modifications of mitochondrial proteins.

Protein	Function	Oxidative Modifications	Consequences	Ref.
Aconitase	TCA cycle	Fe-S cluster oxidation, carbonylation	TCA block, increased ROS	[107,108,109,110]
α-Ketoglutarate dehydrogenase	TCA cycle	Carbonylation of E1/E2 subunits, glutathionylation of lipoic acid, Nitration	Decreased NADH, enhanced ROS	[111,112,113]
Malate dehydrogenase	TCA cycle	Carbonylation (studied in plants)	Reduced TCA flux and NADH regeneration	[108]
MnSOD	Antioxidant enzyme	Tyrosine oxidation, carbonylation, dimer crosslinking (studied in plants)	Loss of superoxide dismutation, higher O_2_^•−^/H_2_O_2_	[108]
Complex I subunits (e.g., NDUFS1, NDUFV1, ND subunits)	ETC	Cys sulfenation/sulfination, carbonylation, flavin oxidation	Impaired electron transfer, increased superoxide (esp. RET), assembly defects	[108,114]
Complex II (SDHA/SDHB)	ETC	Carbonylation, Fe-S centre oxidation	Reduced succinate oxidation, impaired electron transfer	[108,114]
Complex III (cytochrome b, core proteins)	ETC	Dityrosine crosslinks, carbonylation	Blocked Q-cycle, elevated ROS at Qo site	[108,114]
Complex IV (COX subunits I–IV)	ETC	Carbonylation, Cys/Met oxidation	Lower O_2_ reduction capacity, bioenergetic failure	[108,115]
ATP synthase	OXPHOS	Carbonylation, Cys oxidation	Decreased ATP synthesis	[108,115]
VDAC1	Channel	Cys sulfenic/sulfonic acids, carbonylation	Reduced metabolite and Ca^2+^ flux	[116,117]
ANT	nucleotide carrier	Cys oxidation, carbonylation	Impaired ADP/ATP exchange, increased leak/uncoupling	[118,119,120,121]
MCU complex proteins	Ca^2+^ uniporter	S-glutathionylation	higher Ca^2+^ uptake rate, elevated ROS	[122]
Tom20	Import machinery	Cys oxidation, disulfide	Pyroptosis	[123]
HSP60	Chaperone	Carbonylation, Met sulfoxidation	Reduced ROS	[124]
mtHSP70	Chaperone	Cys oxidation	Altered client selection	[125]
OPA1	Fusion protein	Cys oxidation, disulfides	Induction of mitochondrial fusion	[126]
Mitofusins	Fusion	Cys oxidation, crosslinking	Induction of mitochondrial fusion	[126]

**Table 2:** An overview of the glycosylated mitochondrial proteins.

Protein	Glycosylation	Consequences	Ref.
Pyruvate dehydrogenase E1α (PDHA1)	N-linked	Regulates TCA flux entry, potential assembly/trafficking role	[163]
NDUFS3 (Complex I subunit)	N-linked	Complex I assembly/stability, electron transfer modulation	[163]
ADP/ATP translocase (ANT1/ANT2)	N-linked	Enhanced mitochondrial import or stability; possible regulation of nucleotide exchange	[163]
ATP synthase subunit d (ATP5H)	N-linked (WGA-binding)	ATP synthase assembly, cristae organisation	[163]
ATP synthase OSCP (ATP5PO)	N-linked (WGA-binding)	ATP synthase regulation, oligomycin sensitivity	[163]
MRS2 (Mg^2+^ transporter)	N-linked (glycan-dependent)	Reduces rapid Mg^2+^ influx, balances aerobic/anaerobic metabolism	[164]
VDAC1	O-GlcNAc (dynamic)	Modulates permeability, Ca^2+^ flux	[165]
Complex I subunits (NDUFV1, NDUFS1)	O-GlcNAc (multiple sites)	Electron transfer rate, ROS production	[166]
Complex IV COX IV	O-GlcNAc	Respiratory chain activity modulation	[166]

## Data Availability

No new data were created or analyzed in this study. Data sharing is not applicable to this article.
